# Interacting Quantum Atoms—A Review

**DOI:** 10.3390/molecules25174028

**Published:** 2020-09-03

**Authors:** José Manuel Guevara-Vela, Evelio Francisco, Tomás Rocha-Rinza, Ángel Martín Pendás

**Affiliations:** 1 Institute of Chemistry, National Autonomous University of Mexico, Circuito Exterior, Ciudad Universitaria, Delegación Coyoacán C.P., Mexico City 04510, Mexico; jmguevarav@gmail.com (J.M.G.-V.); tomasrocharinza@gmail.com (T.R.-R.); 2 Department of Analytical and Physical Chemistry, University of Oviedo, E-33006 Oviedo, Spain; evelio@uniovi.es

**Keywords:** energy partition, IQA, bonding analysis

## Abstract

The aim of this review is threefold. On the one hand, we intend it to serve as a gentle introduction to the Interacting Quantum Atoms (IQA) methodology for those unfamiliar with it. Second, we expect it to act as an up-to-date reference of recent developments related to IQA. Finally, we want it to highlight a non-exhaustive, yet representative set of showcase examples about how to use IQA to shed light in different chemical problems. To accomplish this, we start by providing a brief context to justify the development of IQA as a real space alternative to other existent energy partition schemes of the non-relativistic energy of molecules. We then introduce a self-contained algebraic derivation of the methodological IQA ecosystem as well as an overview of how these formulations vary with the level of theory employed to obtain the molecular wavefunction upon which the IQA procedure relies. Finally, we review the several applications of IQA as examined by different research groups worldwide to investigate a wide variety of chemical problems.

## 1. Chemical Interactions and Energy Decompositions

If any intrinsic value is to be given to theoretical chemistry beyond that of prediction and thus of its ability to become an in silico alternative to experimental labour, this is its being invaluable for understanding. Chemists propose new synthetic avenues and design new materials according to mental models that owe much to quantum chemistry. Among these, the orbital paradigm has had enormous influence, and the standard textbook classification of chemical interaction types is rooted in it. However, we should not forget that the very phrasing “*chemical interaction*” implies the existence of at least two independent, isolated entities that *interact*, and that this, strictly speaking, is intrinsically forbidden in quantum mechanics, for quantum mechanical systems are non-separable in nature. Once allowed to interact, two systems *A* and *B* become entangled.

The whole of chemical thinking is then rooted on methods designed to approximately separate a fully interacting, non-separable quantum mechanical system into chemically meaningful interacting entities. When chemists talk about redox reactions, electrons are imagined to flow from one of these interacting entities to another. Similar conceptual leaps are involved when pushing arrows or when dipole-dipole interactions are invoked among interacting molecules. In particular, when coming down to energies, any quantitative assessment involves one kind or another of the so-called energy decomposition analyses (EDAs). According to Stones and Hayes [1], an EDA should provide a meaningful partition of the energy into physical terms, lead to total energies in agreement with those obtained in global calculations, and be applicable to wide range of computational or physical conditions. Although many methods have been proposed over the years, most fail to satisfy all of these general criteria.

One of the most difficult to satisfy requirements is the validity of a given EDA at both the short and long range distance regimes. This should be clear to every chemist: the language spoken when talking about *chemical bonds*, full of orbital-related words like covalency, ionicity, π-backdonation, hyperconjugation, and so forth, is rather different from that we talk when describing intermolecular interactions, now stuffed with terms like dispersion, induction, exchange-repulsion among others. This simply reflects the inability of the approximations used in one realm to cross the border that separates them from the other. The approximations are simply incompatible with each other. For instance, the multipolar expansion that lies behind the long-range language simply breaks down at short distances. It is also useless to use perturbation theory, the basis of one of the most accurate methods to obtain intermolecular interaction energies (symmetry adapted perturbation theory, SAPT [2]), to examine the formation of the bond in N${}_{2}$. Similarly, it is not obvious at all how to use the short-range orbital language for very weak interactions.

The history of EDAs is linked to the field of intermolecular interactions. Forces upon molecules were initially studied by understanding full separability among them: a set of separated moieties with a well-defined set of nuclei and electrons and thus isolated Hamiltonians that were allowed to interact via a small electrostatic perturbation. For two interacting entities *A* and *B*, described by isolated Hamiltonians and eigenstates $H^{A}|i^{A}\rangle =E_{i}^{A}|i^{A}\rangle$ and $H^{B}|i^{B}\rangle =E_{i}^{B}|i^{B}\rangle$ so that $H^{0}=H^{A}+H^{B}$,

(1) $$H^{\prime }=-\sum_{i\in A,\beta \in B}\frac{Z_{\beta }}{r_{i\beta }}-\sum_{j\in B,\alpha \in A}\frac{Z_{\alpha }}{r_{j\alpha }}+\sum_{i\in A,j\in B}\frac{1}{r_{ij}}.$$

Notice that $H^{\prime }$ is only an electron perturbation operator. The new nuclear repulsion terms appearing after interaction of *A* and *B* are just additive constants under the Born-Oppenheimer approximation. Blind use of, for instance, a Hartree product for the AB system followed by Rayleigh-Schrödinger theory, violates antisymmetry and is, in principle, forbidden. Much effort has been devoted to solve this problem, and although no unique solution exists, SAPT [2] has become the *de facto* modern standard to solve this problem.

In the very long range regime, anyway, one may use that the partial overlap

(2) $$\int d1\;\Psi^{A}\left(1,2,\ldots ,N_{A}\right)\Psi^{B}\left(1,2^{\prime },\ldots ,N_{B}^{\prime }\right)$$

decays exponentially to neglect it, justifying the plain use of a Hartree product and leading to the so-called polarisation approximation. With this, the states of the subsystem are eigenstates of $H^{0}$, $H^{0}|i^{A}j^{B}\rangle =\left(E_{i}^{A}+E_{j}^{B}\right)|i^{A}j^{B}\rangle =E_{ij}^{0}|i^{A}j^{B}\rangle$. If we ignore degeneracies and take the ground states as $|0^{A}\rangle ,|0^{B}\rangle$, the first and second order corrections to the energy are given by [3]

(3) $$\begin{array}{ccc} E_{00}^{1} & = & \langle 0^{A}0^{B}|H^{\prime }|0^{A}0^{B}\rangle \\ E_{00}^{2} & = & \sum_{ij\neq 00}\frac{\langle 0^{A}0^{B}|H^{\prime }|i^{A}j^{B}\rangle \langle i^{A}j^{B}|H^{\prime }|0^{A}0^{B}\rangle }{E_{ij}^{0}-E_{00}^{0}}. \end{array}$$

Notice that in the standard long-range EDAs the intervening molecules are unperturbed, a condition that cannot be assumed at short distances. The terms in Equation (3) have simple interpretations. $E_{00}^{1}$ is the expectation value of the intermolecular electrostatic perturbation, and is thus known as the electrostatic energy, $E_{elstat}$. The multipolar expansion of $1/r_{12}$ is also usually invoked, so that $E_{elstat}$ is divided into multipole-multipole terms $Q_{l_{a}}Q_{l_{b}}/R_{AB}^{l_{a}+l_{b}+1}$, where the $Q_{l}$ are the unperturbed molecular charge multipoles.

The second order corrections include the classical induction, when one of the i,j indices in Equation (3) is 0, and the dispersion terms, when both are non-zero: $E_{00}^{2}=E_{ind}^{A}+E_{ind}^{B}+E_{disp}^{AB}$. Notice that non-overlapping densities are assumed in all these approximations. Even though the multipolar expansion converges, it may tend to incorrect limits as $R_{AB}$ decreases. Inclusion of antisymmetry effects via SAPT introduces a kind of doubling of each perturbation term, much as the Coulomb integrals have to be supplemented by exchange ones in going from Hartree to Hartree-Fock theory. The first order exchange term, for instance, is called exchange-repulsion, and takes into account Pauli’s destabilisation between closed-shell fragments.

A second category of approaches to deal with energy decomposition is based on applying the general computational chemistry machinery on the AB supermolecule, so that $E_{int}=E^{AB}-E^{A}-E^{B}$ is obtained by subtraction. This approach is plagued with accuracy issues, most of them related to the basis set superposition error (BSSE) [4] and the size-consistency problem of most low-cost high level electron correlation electronic structure methods. Standard protocols, like the counterpoise correction [5], have been devised to correct for them. If we choose to ignore these problems, the issue sets in how to decompose a plain interaction energy $E_{int}$. Two families of methods have been devised to cope with this. Either the A,B partition is performed in Fock (orbital) space, or it is done in real space.

The first route is much more common than the second, and most of the methods that use it go back to the Kitaura and Morokuma (KM) decomposition [6]. For a single determinant approximation, a set of steps are imagined that take us from the isolated fragments to the interacting ones, and for which calculations can be performed. First, the free monomers are distorted from the *in vacuo* to the interacting geometry. The energetic cost of this process is called preparation energy, $E_{prep}$. Then, the electrostatic energy $E_{elstat}$ due to the interaction of overlapping densities is obtained. This is done by avoiding mixing of *A* and *B* primitives in the original Hartree product. An antisymmetrised determinant from unperturbed orbitals is also evaluated, from which the exchange energy $E_{X}$ is obtained, and by subtraction from the final state the charge-transfer energy $E_{CT}$ is finally obtained. Versions in which blocks of the Fock operator were successively suppressed were also proposed.

KM has evolved, and a method inspired on it that has been used very much over the years, usually known as EDA, based on older Ziegler-Rauk decompositions [7] was proposed by Bickelhaupt and Baerends [8]. It can be applied easily in DFT calculations. In EDA, the fragments are first prepared ($E_{prep}$), and their standard electrostatic interaction is obtained $E_{elstat}$. Then, an antisymmetric HF or Kohn-Sham (KS) determinant is formed. The difference in energy of this with respect to the fragments is called the Pauli energy, $\Delta E_{Pauli}$. Finally, the relaxation energy that takes this antisymmetrised system to the interacting complex is called orbital relaxation energy, $\Delta E_{orb}$. Overall,

(4) $$E_{int}=E_{prep}+E_{elstat}+\Delta E_{Pauli}+\Delta E_{orb}.$$

Other popular methods like the LMOEDA [9] approach uses a mixture of a KM-like decomposition and a non-orthogonal treatment. A different, although related, orbital EDA is the Natural Energy Decomposition analysis (NEDA) of Glendening and Streitwiseser [10].

All these, and many other methods, rely on reference states that have no physical meaning, and thus depend on them. When applied to the short range regime to understand intramolecular chemical bonding, for instance, EDA may be obtained for a whole set of ways to partitioned the AB system into fragments. This has been used profitably by some groups to define the most chemically appealing bonding model for a given interaction, for instance, but is obviously not satisfying from the conceptual point of view.

A way to avoid these and other problems is to turn to orbital invariant objects to define energy partitions. These are easy to construct from reduced density matrices (RDMs), which can be defined either in real or in momentum space. Provided that chemists think of molecules residing in the first, not in the second space, methods taking profit of orbital invariance have usually been defined for spatial RDMs, and are called real space energy partitions. In a way, the partitioning problem is now changed to that of how to define our interacting objects, be them atoms or molecules, in space, that is, to how to partition space into atoms or chemical fragments. Out of the possibilities that have been devised, we will basically use the partition provided by the Quantum Theory of Atoms in Molecules (QTAIM) of Bader and coworkers [11].

## 2. The Spatial or Real Space Point of View

An appealing alternative to both perturbation theory and other supermolecular EDAs based on either arbitrarily chosen references or ad hoc approximations is the use of orbital invariant quantities. These are scalar or vector fields that can be obtained as the expectation value of measurement operators. By construction, they do depend neither on external references nor on the specific electronic structure methodology. A well known example is the electron density ρ(r), which is routinely obtained via X-ray diffraction experiments [12] and that can be defined as the expectation value of an electron position measurement operator:

(5) $$\rho \left(r\right)=\langle \Psi |\hat{\rho }\left(r\right)|\Psi \rangle ,$$

where $\hat{\rho }\left(r\right)=\sum_{i=1}^{N}\delta \left(r-r_{i}\right)$ sums over all spatial electron coordinates. It is easily shown that

(6) $$\rho \left(r_{1}\right)=N\int \Psi^{*}\left(x_{1},x_{2},\ldots ,x_{N}\right)\Psi \left(x_{1},x_{2},\ldots ,x_{N}\right)\;d\sigma_{1}dx_{2}\ldots dx_{N},$$

where $x_{i}\equiv r_{i}\sigma_{i}$ gathers the spatial ($r_{i}$) and spin ($\sigma_{i}$) coordinates of electron *i*.

If we choose the momentum representation we can also find the momentum density, which is not so much used in Chemistry. We will not comment in this review on momentum space quantities anymore.

This approach can be easily generalised to consider the density of electron tuples: pairs, trios, and so forth, leading to the so-called reduced density matrix (RDM) formalism. In general, the *n*-th order RDM (nRDM) is defined as [13]

(7) $$\rho_{n}\left(x_{1}^{\prime },\ldots ,x_{n}^{\prime };x_{1},\ldots ,x_{n}\right)=\frac{N!}{(N-n)!}\int \Psi^{*}\left(x_{1}^{\prime },x_{2}^{\prime },\ldots ,x_{N}^{\prime }\right)\Psi \left(x_{1},x_{2},\ldots ,x_{N}\right)\;dx_{n+1}\ldots dx_{N}.$$

Spinless variants are simply obtained by summing up the spin degrees of freedom of the 1,…,n electrons. Their diagonal parts, when $x_{i}^{\prime }=x_{i}$ ∀i∈[1,n] are called *n*-th order reduced densities (nRDs), and provide the probability densities of finding electron tuples at particular points in space. As we will see in more detail, the electronic molecular energy depends only on the 1RDM and the 2RD: $E=\mathrm{Tr}\left(h\rho_{1}\right)+1/2\mathrm{Tr}\left(\rho_{2}/r_{12}\right)$.

The essential issue of spatial or real space energy partitions is how to divide the physical space $R^{3}$ into a given number of domains *m* endowed with a relevant physical meaning. From a purely mathematical point of view, a fairly general way of doing this is to define a function $f\left(r\right)=\sum_{A}^{m}\omega_{A}\left(r\right)$, such that f(r)=1 ∀r. Different choices of $\omega_{A}\left(r\right)$ provide different partitions of the real space. With this simple definition it is evident that an arbitrary function of r (say ρ(r)) can be decomposed into as many contributions as the number of terms in the summation that defines f(r), that is, $\rho \left(r\right)=\sum_{A}^{m}\rho_{A}\left(r\right)$, where $\rho_{A}\left(r\right)=\rho \left(r\right)\omega_{A}\left(r\right)$. By extension, an arbitrary function $F(r_{1},\ldots ,r_{n})$ can always be put in the form

(8) $$\begin{array}{ccc} F(r_{1},\ldots ,r_{n}) & = & \sum_{AB\cdots }^{m}\omega_{A}\left(r_{1}\right)\omega_{B}\left(r_{2}\right)\cdots F\left(r_{1},\ldots ,r_{n}\right)\\ & = & \sum_{AB\cdots }^{m}F_{AB\cdots }\left(r_{1},\ldots ,r_{n}\right). \end{array}$$

In general, there are $m^{n}$ functions $F_{AB\cdots }$. However, in the cases we are interested in, there exist relations between these functions that makes the number of them that is independent less than $m^{n}$. For instance, when $F(r_{1},r_{2})$ is the spinless 2RDM $\rho_{2}\left(r_{1},r_{2}\right)$, one has $\rho_{2,BA}\left(r_{1},r_{2}\right)=\rho_{2,AB}\left(r_{1},r_{2}\right)$, and the number of independent functions is only m(m+1)/2.

Although infinite definitions of f(r) satisfying f(r)=1 are possible, only some of them based in a partition of the real space endowed with a deep physical meaning, deserve to be explored and have been used to date. It should be noted first that the physical space ($R^{3}$) can be divided into fuzzy or exhaustive regions. Among the former, the so-called Hirshfeld partition and its different versions, References [14,15] among others [16,17,18,19], has received some attention so far. In it, every subindex *A* in $\omega_{A}\left(r\right)$ refers to an atom of the molecule, and these atomic functions are defined based on the electron atomic densities as $\omega_{A}\left(r\right)=\rho_{A}^{0}\left(r\right)/\sum_{A}\rho_{A}^{0}\left(r\right)$, where $\rho_{A}^{0}\left(r\right)$ is the *in vacuo* density of atom *A* or the density of this atom modified by its molecular environment. As other fuzzy partitions, Hirshfeld-like divisions of $R^{3}$ have the characteristic that each $\omega_{A}\left(r\right)$ takes a value close to unity when the point r is close to the nucleus of atom *A* and decreases progressively as it moves away from it. Another widely used definition of $\omega_{A}\left(r\right)$ is that proposed by Becke [16] to simplify the numerical integrations of Density Functional Theory (DFT). Becke’s partition divides the physical space into atomic regions that resemble fuzzy Voronoi polyhedron. The size of each atomic domain can be adjusted by using an effective radius $R_{A}$ for each of the atoms of the molecule [16]. These $R_{A}$’s can be chosen in different ways [17]. Finally, it is worth mentioning the choice for $\omega_{A}\left(r\right)$ proposed by Fernández Rico et. al. [19], that determines $\rho_{A}\left(r\right)$ following a minimal deformation criterion for every two-centre contribution to the electron density ρ(r).

Contrary to fuzzy partitions, exhaustive partitions of $R^{3}$ have $\omega_{A}\left(r\right)=1$ when $r\in \Omega_{A}$ and $\omega_{A}\left(r\right)=0$ when $r\notin \Omega_{A}$, where $\Omega_{A}$ is a given domain or region of $R^{3}$. Among these, the $\Omega_{A}$’s induced by the topology of a scalar field that exhaustively partition the real space, such as the molecular density ρ(r), or the electron localisation function (ELF) of Becke and Edgecombe [20] stand out on their own merits, have been and continue to be widely used. Let us very briefly state that the 3D maxima or minima of any well-behaved scalar field f(r) induce a topological exhaustive partition of the physical space into non-overlapping regions. These are the basins of attraction (or repulsion) of the maxima (minima), built as the geometrical locus of all points in space whose gradient field lines end (start) at each maximum (minimum). It is easy to show that the surfaces separating the different basins are local zero-flux surfaces: ∇f(r)·n(r)=0, where n is the exterior normal vector at each point r of the surface. Obviously, a local zero-flux surface is also a global zero-flux one: $\oint_{S}\nabla f\left(r\right)\cdot dS=0$.

The partition of $R^{3}$ based on the topology of ρ(r) is the key element that supports the Quantum Theory of Atoms in Molecules (QTAIM) developed by Prof. Bader and his collaborators [11], and the starting point of the interacting quantum atoms method (IQA) [21,22], whose description is the essential objective of this work. Each $\Omega_{A}$ is then identified with an atomic basin, that includes the nucleus of the atom plus an open region around it that contains an average number of electrons that is determined by the electron density ρ(r), whose topological analysis, in turn, defines $\Omega_{A}$.

## 3. The IQA Methodology

The interacting quantum atoms (IQA) method is an orbital invariant energy partition that divides the total energy of a molecule into a sum of atomic self-energies and interaction energies between all the atoms of the molecule. Unlike the orthodox QTAIM, the IQA partition can be performed at any molecular geometry. As a consequence, it can be particularly useful to follow the evolution of arbitrary interatomic or inter-fragment interaction energies, and intra-fragment energies as well, along the path of a chemical reaction. Notice, however, the application of IQA has been limited by its compraratively much worse computational scaling with respect to other EDA schemes.

Succinctly, since extending on this topic would lead to a considerably more extense discussion, at stationary points of potential energy surfaces the virial theorem holds and the total molecular kinetic (*T*) and potential (V) energies get intertwinned, 2T+V=0, so that T+V=E=−T. Within the QTAIM, the atomic virial theorem [11] can then be exploited to define an atomic energy for each quantum atom *A* as $E^{A}=-T^{A}$ that is fully additive: $E=\sum_{A}E^{A}$. These virial energies have been exploited successfully, but face considerable problems outside stationary geometries. In these general cases, the virial theorem reads $2T+V=\sum_{\alpha }R_{\alpha }\cdot F_{\alpha }$, where α runs over the nuclei, and R and F stand for the nuclear positions and their Hellmann-Feynman forces. This sum is usually called the nuclear virial, and there is no unique, simple way to divide it or split it into origin independent atomic terms. This difficulty is at the root of the more general perspective provided by IQA.

The starting point to apply IQA are the first order, $\rho_{1}\left(r_{1};r_{1}^{\prime }\right)$, and (diagonal) second order, $\rho_{2}\left(r_{1},r_{2}\right)$, RDMs of the system. From the general expression given in Equation (7) they are given as

(9) $$\begin{array}{ccc} \rho_{1}\left(r_{1};r_{1}^{\prime }\right) & = & N\int \Psi^{⋆}\left(x_{1}^{\prime },x_{2},\ldots ,x_{N}\right)\Psi \left(x_{1},x_{2},\ldots ,x_{N}\right)\;d\sigma \;dr_{i\geq 2}, \end{array}$$

(10) $$\begin{array}{ccc} \rho_{2}\left(r_{1},r_{2}\right) & = & N\left(N-1\right)\int \Psi^{⋆}\left(x_{1},x_{2},\ldots ,x_{N}\right)\Psi \left(x_{1},x_{2},\ldots ,x_{N}\right)\;d\sigma \;dr_{i\geq 3}, \end{array}$$

where $\sigma =\sigma_{1},\cdots ,\sigma_{N}$ refers to the spin coordinates of all the electrons, and $dr_{i\geq n}$ indicates spatial integration from electron *n* to *N*. Actually, Ψ(1,N) is not strictly necessary in IQA. All that is required are $\rho_{1}\left(r_{1};r_{1}^{\prime }\right)$ and $\rho_{2}\left(r_{1},r_{2}\right)$, regardless of how these have been obtained (*vide infra*). It is even possible to use electronic densities from difraction experiments as inputs in the IQA calculations [23,24]. Thanks to this, IQA can deal with electronic structure methods that give rise to a wavefunction, such as the Hartree-Fock (HF), the complete active space (CAS), or the full Interaction of Configurations (full-CI) methods, for which $\rho_{1}\left(r_{1};r_{1}^{\prime }\right)$ and $\rho_{2}\left(r_{1},r_{2}\right)$ can be derived, but also with other methods where the wavefunction is not available but in which more or less accurate forms for $\rho_{1}\left(r_{1};r_{1}^{\prime }\right)$ and $\rho_{2}\left(r_{1},r_{2}\right)$ can be found. Among the latter, both the Coupled Cluster (CC) method [25], due to the very high accuracy results it generates in medium and small systems, and the Møller-Plesset perturbative approach should be highlighted.

Leaving aside the errors associated with the three- and hexa-dimensional numerical integrations that are characteristic of the method, IQA recovers exactly the total energy of the molecular system under study. None of the energy components is calculated by making approximations of any kind in order to accelerate these numerical integrations. Given the $\rho_{1}\left(r_{1};r_{1}^{\prime }\right)$ and $\rho_{2}\left(r_{1},r_{2}\right)$ RDMs, and assuming negligible integration errors, the IQA energy components add to the total molecular energy. Of course, IQA offers also the possibility of calculating in a simplified form some inter-atomic interactions (specifically Coulomb-type interactions and exchange interactions between atoms or fragments far enough apart), but only in an optional way, particularly in those cases where the exact calculation would be prohibitive or for purely comparative purposes.

A remarkable feature of the IQA methodology is its orbital invariance. Most times, $\rho_{1}\left(r_{1};r_{1}^{\prime }\right)$ and $\rho_{2}\left(r_{1},r_{2}\right)$ are built with the canonical molecular orbitals (MO) of the system. However, it is also possible to replace them with the corresponding natural orbitals or, even more interesting, with real space localised MOs, obtained from the canonical MOs with one of the different localisation schemes existing in the literature. This last possibility is very attractive in correlated calculations with a high number of orbitals. In these cases it is essential to truncate $\rho_{1}\left(r_{1};r_{1}^{\prime }\right)$ and $\rho_{2}\left(r_{1},r_{2}\right)$ by eliminating some of them: the less localised some MOs are in two atoms of the system (say A and B) the less the interaction between both atoms will be affected by eliminating these orbitals from the complete list used to construct the density matrices.

Although until recently the IQA methodology had been applied to molecular systems in the ground electronic state, there is nothing preventing its application in excited electronic states [26]. Very recently, the black-box equation-of-motion (EOM) coupled-cluster singles and doubles (CCSD) approximation has been used to construct $\rho_{1}\left(r_{1};r_{1}^{\prime }\right)$ and $\rho_{2}\left(r_{1},r_{2}\right)$ and to use the IQA method to dissect excitation energies into atomic and interatomic contributions of molecular clusters such as (H${}_{2}$O)${}_{2}$, getting very valuables insights in photochemistry.

In a similar way, even though IQA requires, in principle, the availability of $\rho_{2}\left(r_{1},r_{2}\right)$, whatever the electronic structure method used to generate it, a proposal has been formulated such that IQA can be applied with Kohn-Sham determinants (formally, single-determinant pseudo-wavefunctions) obtained from DFT calculations. This fact opens up the possibility of performing IQA calculations on systems larger than those analysed to date.

After this general preamble, we will present in Section 3.1 the general IQA formalism, paying a special emphasis on the presentation of the energetic components in which IQA divides the total energy of an arbitrary molecule and in how these are grouped together into quantities endowed with a clear physical meaning. In Section 3.2, we will briefly show the specific peculiarities of the IQA method with the different forms that have been used so far for the 1RDM and 2RDM. Finally, the algorithms used to carry out the complex three- and hexa-dimensional numerical integrations are considered in Section 3.3.

### 3.1. The Iqa Energy Partition

The total electronic energy of a molecule, within an arbitrary wavefunction-based electronic structure method different from DFT, may be written under the usual Coulomb Hamiltonian as

(11) $$\begin{array}{c} E=h+V_{ee}+V_{nn}=\int \hat{h}\rho_{1}\left(r_{1};r_{1}^{\prime }\right)dr_{1}\\ +\frac{1}{2}\;\int \int \;dr_{1}dr_{2}\frac{\rho_{2}\left(r_{1},r_{2}\right)}{r_{12}}+V_{nn}. \end{array}$$

The prime superscript in $\rho_{1}$ is removed prior to integration, but after $\hat{h}$ acts on it. This operator gathers all monoelectronic terms: electronic kinetic energy, $\hat{T}$, and electron–nuclear attraction, ${\hat{V}}_{en}$. The term $V_{ee}$ is the interelectron repulsion, and the last one is the internuclear repulsion energy, $V_{nn}=\sum_{A>B}Z^{A}Z^{B}R_{AB}^{-1}$. Considering that $\rho_{2}\left(r_{1},r_{2}\right)$ can be naturally partitioned into Coulomb and exchange-correlation (xc) contributions,

(12) $$\begin{array}{ccc} \rho_{2}\left(r_{1},r_{2}\right) & = & \rho_{2}^{J}\left(r_{1},r_{2}\right)+\rho_{2}^{xc}\left(r_{1},r_{2}\right), \end{array}$$

(13) $$\begin{array}{ccc} & = & \rho \left(r_{1}\right)\rho \left(r_{2}\right)+\rho_{2}^{xc}\left(r_{1},r_{2}\right), \end{array}$$

$V_{ee}$ can always be split as $V_{ee}=V_{cl}+V_{xc}$, where:

(14) $$\begin{array}{ccc} V_{cl} & = & \frac{1}{2}\;\int \int \;dr_{1}dr_{2}\frac{\rho_{2}^{J}\left(r_{1},r_{2}\right)}{r_{12}}\;\mathrm{and} \end{array}$$

(15) $$\begin{array}{ccc} V_{xc} & = & \frac{1}{2}\;\int \int \;dr_{1}dr_{2}\frac{\rho_{2}^{xc}\left(r_{1},r_{2}\right)}{r_{12}}, \end{array}$$

are the total classical and xc molecular energies, respectively. All the integrations in the above expressions are extended to the whole three dimensional space. Using the definitions given in Section 2 and, in particular, Equation (8), *E* is exactly given by

(16) $$\begin{array}{ccc} E & = & \sum_{A}h_{A}+\frac{1}{2}\sum_{A}\sum_{B}V_{ee}^{AB}+V_{nn},\;\mathrm{where} \end{array}$$

(17) $$\begin{array}{ccc} h_{A} & = & \int \omega_{A}\left(r\right)\hat{h}\rho_{1}\left(r;r^{\prime }\right)dr,\;\mathrm{and} \end{array}$$

(18) $$\begin{array}{ccc} V_{ee}^{AB} & = & \frac{1}{2}\sum_{A,B}\;\int \int \;dr_{1}dr_{2}\frac{\omega_{A}\left(r_{1}\right)\omega_{B}\left(r_{2}\right)\rho_{2}\left(r_{1},r_{2}\right)}{r_{12}}. \end{array}$$

We must remember at this point that $\omega_{A}\left(r\right)=1$ if $r\in \Omega_{A}$ and $\omega_{A}\left(r\right)=0$ if $r\notin \Omega_{A}$, where $\Omega_{A}$ is the basin associated to atom *A* according to QTAIM. With these premises, it is only necessary to group the different energy terms of Equation (16) into two categories: (1) those that only involve the particles (nucleus plus electrons) within a single atomic basin $\Omega_{A}$ and (2) those that involve interactions between the particles from two different regions $\Omega_{A}$ and $\Omega_{B}$. The result is the main equation of the IQA energy partition (to simplify the notation $\Omega_{A}$ is replaced by *A* from now on):

(19) $$\begin{array}{ccc} E & = & \sum_{A}E_{\mathrm{self}}^{A}+\sum_{A>B}E_{\mathrm{int}}^{AB} \end{array}$$

(20) $$\begin{array}{ccc} E_{\mathrm{self}}^{A} & = & T^{A}+V_{\mathrm{ne}}^{AA}+V_{\mathrm{ee}}^{AA} \end{array}$$

(21) $$\begin{array}{ccc} E_{\mathrm{int}}^{AB} & = & V_{\mathrm{ne}}^{AB}+V_{\mathrm{ne}}^{BA}+V_{\mathrm{ee}}^{AB}+V_{\mathrm{nn}}^{AB}. \end{array}$$

$E_{\mathrm{self}}^{A}$ is the self-energy of the atomic basin $\Omega_{A}$, and includes the kinetic energy of the electron density inside $\Omega_{A}$,

(22) $$T^{A}=\int_{A}\hat{T}\rho_{1}\left(r;r^{\prime }\right)dr,$$

the attraction between the nucleus and electrons in $\Omega_{A}$,

(23) $$V_{\mathrm{ne}}^{AA}=-Z^{A}\int_{A}dr\frac{\rho (r)}{|r-R^{A}|},$$

and the repulsion between the electrons in $\Omega_{A}$,

(24) $$V_{\mathrm{ee}}^{AA}=\frac{1}{2}\int_{A}dr_{1}\int_{A}dr_{2}\frac{\rho_{2}\left(r_{1},r_{2}\right)}{r_{12}}.$$

$E_{\mathrm{int}}^{AB}$ is the total interaction energy between the atomic basins $\Omega_{A}$ and $\Omega_{A}$, including the nucleus(A)-nucleus(B), nucleus(A)-electrons(B), nucleus(B)-electrons(A), and electrons(A)-electrons(B) terms, given respectively by:

(25) $$\begin{array}{ccc} V_{\mathrm{nn}}^{AB} & = & Z^{A}Z^{B}R_{AB}^{-1}, \end{array}$$

(26) $$\begin{array}{ccc} V_{\mathrm{ne}}^{AB} & = & -Z^{A}\int_{B}dr\frac{\rho (r)}{|r-R^{A}|}, \end{array}$$

(27) $$\begin{array}{ccc} V_{\mathrm{ne}}^{BA} & = & -Z^{B}\int_{A}dr\frac{\rho (r)}{|r-R^{B}|}, \end{array}$$

(28) $$\begin{array}{ccc} V_{\mathrm{ee}}^{AB} & = & \int_{A}dr_{1}\int_{B}dr_{2}\frac{\rho_{2}\left(r_{1},r_{2}\right)}{r_{12}}. \end{array}$$

Using Equation (12), $V_{ee}^{AB}$ can be split into a classical electron-electron interaction term,

(29) $$V_{\mathrm{ee},\mathrm{cl}}^{AB}=\int_{A}dr_{1}\int_{B}dr_{2}\frac{\rho_{2}^{J}\left(r_{1},r_{2}\right)}{r_{12}},$$

and an xc interaction energy

(30) $$V_{\mathrm{xc}}^{AB}=\int_{A}dr_{1}\int_{B}dr_{2}\frac{\rho_{2}^{xc}\left(r_{1},r_{2}\right)}{r_{12}}.$$

In this way, the total AB interaction energy, $E_{\mathrm{int}}^{AB}$, admits the following more chemical reorganisation

(31) $$E_{\mathrm{int}}^{AB}=V_{\mathrm{cl}}^{AB}+V_{\mathrm{xc}}^{AB},$$

where $V_{\mathrm{cl}}^{AB}$ is the total classical interaction between the atomic basins $\Omega_{A}$ and $\Omega_{B}$,

(32) $$V_{\mathrm{cl}}^{AB}=\int_{A}dr_{1}\int_{B}dr_{2}\frac{\rho_{A}^{T}\left(r_{1}\right)\rho_{B}^{T}\left(r_{2}\right)}{r_{12}},$$

with $\rho_{A}^{T}\left(r\right)=Z^{A}\delta \left(r-R^{A}\right)-\rho \left(r\right)$.

It is important to note that the $V_{\mathrm{cl}}^{AB}$ definition is formally identical to the electrostatic terms in perturbative expansions or in the Energy Decomposition Analysis (EDA) procedure [8]. However, in the IQA method the electron densities $\rho_{A}^{T}\left(r_{1}\right)$ and $\rho_{B}^{T}\left(r_{2}\right)$ do not interpenetrate. This is the source of important numerical differences between EDA-like and IQA classical interactions. We should mention here that, even though $\rho_{A}^{T}\left(r_{1}\right)$ and $\rho_{B}^{T}\left(r_{2}\right)$ do not interpenetrate in IQA, these two densities may be, however, overlapping in the sense to be discussed in Section 3.3. The *overlap* between $\rho_{A}^{T}\left(r_{1}\right)$ and $\rho_{B}^{T}\left(r_{2}\right)$ is the origin of the non-convergence or lack of validity of the well-known multipolar expansion (widely used to determine $V_{\mathrm{cl}}^{AB}$) when atoms *A* and *B* are not far enough from each other [27,28]. As we will see, since this *overlap* is explicitly taken into account, this lack of convergence does not occur in the IQA method.

$V_{\mathrm{xc}}^{AB}$ represents the covalent interaction between atoms *A* and *B* [29,30]. A null value of this term signals the absence of electron exchange between both atoms, and being this exchange the fundamental identity sign of a covalent bond, a zero value of $V_{\mathrm{xc}}^{AB}$ simply means that atoms *A* and *B* are not covalently bonded. Let’s be even clearer and consider a molecule formed only by two atoms *A* and *B* separated by a distance *R* in which the classical interaction is close to −1/R but has $V_{\mathrm{xc}}^{AB}=0$. This simply means that approximately one electron has been permanently transferred from *A* to *B* (or vice versa), but once the electron has been transferred, it is not exchanged any more between both atoms. In other words, there is no fluctuation in the electronic population of either of the two atoms, which is equivalent to say that the covariance of the electron population of both atoms (say $n_{A}$ and $n_{B}$) is zero, $\mathrm{cov}(n_{A},n_{B})=0$. Real space theories of chemical bonding establish that the bond order between *A* and *B* is given by $\delta^{AB}=-2\mathrm{cov}\left(n_{A},n_{B}\right)$ [31,32]. Hence, a double implication exists $\left(V_{\mathrm{xc}}^{AB}=0\right)\Leftrightarrow \left(\delta^{AB}=0\right)$. An algebraic proof of the existing connection between $V_{\mathrm{xc}}^{AB}$ and $\delta^{AB}$ is given in a later subsection.

The atomic self-energy $E_{\mathrm{self}}^{A}$ contains all of the energy contributions that are already present in the isolated atom *A*, and so the free atomic energies, $E_{\mathrm{vac}}^{A}$, are comparable in order of magnitude. The binding energy of the molecule, defined as its total energy *E* minus the sum of all the free atomic energies, $E_{\mathrm{bind}}=E-\sum_{A}E_{\mathrm{vac}}^{A}$, can then be written as

(33) $$\begin{array}{ccc} E_{\mathrm{bind}} & = & \sum_{A}E_{\mathrm{def}}^{A}+\sum_{A>B}E_{\mathrm{int}}^{AB}, \end{array}$$

(34) $$\begin{array}{ccc} & = & \sum_{A}E_{\mathrm{def}}^{A}+\sum_{A>B}\left[V_{\mathrm{cl}}^{AB}+V_{\mathrm{xc}}^{AB}\right]. \end{array}$$

$E_{\mathrm{def}}^{A}$ is the deformation energy of atom *A* and measures the change in energy of this atom in passing from the isolated to the in-the-molecule state. Although we will not pursue here a more detailed analysis of this energy, it is important to say that it can be written in turn as the sum of two components, $E_{\mathrm{def}}^{A}=E_{\mathrm{def}}^{A,\rho }+E_{\mathrm{def}}^{A,ct}$. The first term, always present, is due to the deformation of the atomic density and is positive as long as $E_{\mathrm{vac}}^{A}$ is variationally determined with the same basis later employed in the molecule. This is so because, by definition, the density of the isolated atom *A*, $\rho_{\mathrm{vac}}^{A}\left(r\right)$, minimises $E_{\mathrm{vac}}^{A}$. The second term, $E_{\mathrm{def}}^{A,ct}$, is mainly associated to the electron transfer from atom *A* to the remaining atoms of the molecule or vice versa, and to others effects like spin-recoupling, and so forth. It clearly vanishes in homodiatomic molecules, $A_{2}$, but is different from zero when atom *A* gains or loses electron density when the molecule is formed from the isolated atoms. Usually, it is negative in the first case and positive in the second.

The total interaction energy $E_{\mathrm{int}}^{AB}$ has recently be shown to be a good measure of the in situ bond strength between the atoms *A* and *B* [33]. The sum of these interaction energies for all different pairs of atoms *A* and *B*, $\sum_{A>B}E_{\mathrm{int}}^{AB}$, would be the total atomisation or fragmentation energy of the molecule if the atomic energies are are measured with respect to their in-the-molecule state [33].

All the above equations correspond to a partition of the molecule into atoms. However, they can be easily generalised when the molecule is divided into several groups or fragments, each containing one or more atoms [22]. For instance, calling G and H two generic groups of the molecule, Equations (19) and (21) remain the same if *A* and *B* are replaced by G and H, respectively, and $E_{\mathrm{self}}^{G}$ is equal to (20), with G instead of *A*, and adding a term $V_{nn}^{\mathrm{GG}}=\sum_{\left(A\in G)>(B\in G\right)}Z^{A}Z^{B}R_{AB}^{-1}$ that accounts for the repulsion between the different nuclei of group G, in the event that this group has more than one atom. The remaining contributions to $E_{\mathrm{self}}^{G}$ must be trivially modified as follows: $T^{G}$ is given by Equation (22) with the integration over r extended to $\Omega_{G}=\sum_{A\in G}\Omega_{A}$, that is, to the set of atomic basins of all the atoms that belong to group G, and the intra group nuclei-electrons and electrons-electron energies $V_{ne}^{\mathrm{GG}}$ and $V_{ee}^{\mathrm{GG}}$ are given by

(35) $$V_{\mathrm{ne}}^{\mathrm{GG}}=-\sum_{A\in G}Z^{A}\int_{G}dr\frac{\rho (r)}{|r-R^{A}|},$$

(36) $$V_{\mathrm{ee}}^{\mathrm{GG}}=\frac{1}{2}\int_{G}dr_{1}\int_{G}dr_{2}\frac{\rho_{2}\left(r_{1},r_{2}\right)}{r_{12}}.$$

Similarly, the four energy terms of $E_{\mathrm{int}}^{\mathrm{GH}}$ are given by generalisation of Equations (25)–(28)

(37) $$\begin{array}{ccc} V_{\mathrm{nn}}^{\mathrm{GH}} & = & \sum_{A\in G}\sum_{B\in H}Z^{A}Z^{B}R_{AB}^{-1}, \end{array}$$

(38) $$\begin{array}{ccc} V_{\mathrm{ne}}^{\mathrm{GH}} & = & -\sum_{A\in G}Z^{A}\int_{H}dr\frac{\rho (r)}{|r-R^{A}|}, \end{array}$$

(39) $$\begin{array}{ccc} V_{\mathrm{ne}}^{\mathrm{HG}} & = & -\sum_{B\in H}Z^{B}\int_{G}dr\frac{\rho (r)}{|r-R^{B}|}, \end{array}$$

(40) $$\begin{array}{ccc} V_{\mathrm{ee}}^{\mathrm{GH}} & = & \int_{G}dr_{1}\int_{H}dr_{2}\frac{\rho_{2}\left(r_{1},r_{2}\right)}{r_{12}}. \end{array}$$

The last equation when $\rho_{2}$ is replaced by $\rho_{2}^{xc}$ gives $V_{\mathrm{xc}}^{\mathrm{GH}}$. Finally, the total classical interaction between the groups G and H is given by

(41) $$V_{\mathrm{cl}}^{\mathrm{GH}}=\int_{G}dr_{1}\int_{H}dr_{2}\frac{\rho_{G}^{T}\left(r_{1}\right)\rho_{H}^{T}\left(r_{2}\right)}{r_{12}},$$

where $\rho_{G}^{T}\left(r\right)=\sum_{A\in G}Z^{A}\delta \left(r-R^{A}\right)-\rho \left(r\right)$.

### 3.2. Iqa from Different Electronic Structure Approximations

The equations presented in the previous subsection constitute the essential core of the IQA methodology and, with very slight changes, were already present in the original formulation of the method [22,34]. Many of the improvements in recent years have consisted of expanding the set of types of wavefunctions with which it is possible to apply these equations. In particular, as it is evident from Equation (11), the more exact $\rho_{1}$ and $\rho_{2}$, the better the quality and plausibility of the results obtained for the different energy components. Even though RDMs of a given method are not of higher quality to others already incorporated in the IQA methodology, no doubt that the formalism is enriched as it is capable of dealing with an expanded set of forms for $\rho_{1}$ and $\rho_{2}$, coming from different types of electronic structure methods. Regardless the method used, $\rho_{1}$ and $\rho_{2}$ in a given basis of molecular orbitals $\phi_{i}$ (usually the canonical HF orbitals) are written in the form [35]

(42) $$\rho_{1}\left(r;r^{\prime }\right)=\sum_{pq}D_{pq}\phi_{p}^{⋆}\left(r^{\prime }\right)\phi_{q}\left(r\right)$$

(43) $$\rho_{2}\left(r_{1},r_{2}\right)=\sum_{pqrs}d_{pqrs}\phi_{p}^{⋆}\left(r_{1}\right)\phi_{q}\left(r_{1}\right)\phi_{r}^{⋆}\left(r_{2}\right)\phi_{s}\left(r_{2}\right).$$

#### 3.2.1. Densities for Single- and Multideterminantal Wavefunctions

In the simplest mean-field or HF approximation the summations over *p*, *q*, *r*, and *s* in the above two equations (assuming a closed-shell molecule for simplicity) are from 1 to n=N/2. In this approximation, *D* is diagonal, $D_{pq}=n_{p}\delta_{pq}$, where $n_{p}=2$ is the occupation of $\phi_{p}$, and $d_{pqrs}=4\delta_{pq}\delta_{rs}-2\delta_{pr}\delta_{qs}$. Using these expressions of $D_{pq}$ and $d_{pqrs}$ in Equations (42) and (43) we have

(44) $$\rho_{1}^{\mathrm{HF}}\left(r;r^{\prime }\right)=\sum_{p}^{n}n_{p}\phi_{p}^{⋆}\left(r^{\prime }\right)\phi_{p}\left(r\right),\mathrm{and}$$

$$\begin{array}{ccc} \rho_{2}\left(r_{1},r_{2}\right) & = & \rho^{\mathrm{HF}}\left(r_{1}\right)\rho^{\mathrm{HF}}\left(r_{2}\right) \end{array}$$

(45) $$\begin{array}{ccc} & - & \sum_{pq}^{n}2\phi_{p}^{⋆}\left(r_{1}\right)\phi_{q}\left(r_{1}\right)\phi_{p}^{⋆}\left(r_{2}\right)\phi_{q}\left(r_{2}\right), \end{array}$$

(46) $$\begin{array}{ccc} & = & \rho_{2}^{J}\left(r_{1},r_{2}\right)+\rho_{2}^{xc}\left(r_{1},r_{2}\right), \end{array}$$

where $\rho^{\mathrm{HF}}\left(r\right)\equiv \rho_{1}^{\mathrm{HF}}\left(r;r\right)$. At this point, it is relevant to note that, assuming real MOs and defining the basis of pairs of MOs $G_{1}=\phi_{1}\phi_{1}$, $G_{2}=\phi_{2}\phi_{1}$, $G_{3}=\phi_{2}\phi_{2}$, …, $\rho_{2}^{xc}$ acquires the form

(47) $$\rho_{2}^{xc}\left(r_{1},r_{2}\right)=\sum_{i}^{n(n+1)/2}\lambda_{i}G_{i}\left(r_{1}\right)G_{i}\left(r_{2}\right),$$

where $\lambda_{i}=-2$ for i=1,3,6,…, and $\lambda_{i}=-4$ for the remaining values of *i*. As we will see, this *diagonal* form of $\rho_{2}^{xc}$ has important implications related to the numerical integrations of the method.

When $\rho_{1}$ and $\rho_{2}$ come from a multideterminantal wavefunction (say, from a complete active space (CAS) or full interaction of configurations (full-CI) calculation), neither $\rho_{1}$ is diagonal in the canonical basis $\phi_{i}$ (as in Equation (44)) nor $\rho_{2}^{xc}\left(r_{1},r_{2}\right)$ can be written as in Equation (47). In this and other cases to be discussed below, the present implementation of IQA proceeds as follows. Firstly, $\rho_{1}$ is built in the canonical basis, obtaining the *D* matrix, which after being diagonalised leads to

(48) $$\rho_{1}^{\mathrm{CAS}}\left(r;r^{\prime }\right)=\sum_{p}^{m}n_{p}\varphi_{p}^{⋆}\left(r^{\prime }\right)\varphi_{p}\left(r\right),$$

where $\varphi_{p}$ are the natural MOs of the system and $n_{p}$ ($0\leq n_{p}\leq 2$) are their occupation numbers. The upper limit m≥n in Equation (48) is the size of the MO basis used in the calculation.

Regarding $\rho_{2}^{xc}$, its expression in the $\phi_{i}$ basis is analogous to Equation (43) for the complete $\rho_{2}$, that is, (assuming again real MOs)

(49) $$\begin{array}{ccc} \rho_{2}^{xc}\left(r_{1},r_{2}\right) & \;\;=\;\; & \sum_{pqrs}\epsilon_{pqrs}\phi_{p}\left(r_{1}\right)\phi_{q}\left(r_{1}\right)\phi_{r}\left(r_{2}\right)\phi_{s}\left(r_{2}\right) \end{array}$$

(50) $$\begin{array}{ccc} & \;\;\equiv \;\; & \sum_{p\geq q,r\geq s}\eta_{pq,rs}F_{pq}\left(r_{1}\right)F_{rs}\left(r_{2}\right), \end{array}$$

were we have defined $F_{pq}=\phi_{p}\phi_{q}$ and $\eta_{pq,rs}=\epsilon_{pqrs}+\epsilon_{pqsr}\Delta_{rs}+\epsilon_{qprs}\Delta_{pq}+\epsilon_{qpsr}\Delta_{pq}\Delta_{rs}$, with $\Delta_{ab}=0$ if a=b and $\Delta_{ab}=1$ if a≠b. Condensing the pair of indices (p,q) in a single index *i*, and the pair of indices (r,s) in another index *j*, both *i* and *j* varying from 1 to m(m+1)/2, one obtains

(51) $$\rho_{2}^{xc}\left(r_{1},r_{2}\right)=\sum_{ij}\eta_{ij}F_{i}\left(r_{1}\right)F_{j}\left(r_{2}\right).$$

η is a symmetric array that can be diagonalised [36]. Calling $\lambda_{i}$ and $G_{i}$ its eigenvalues and eigenvectors, respectively, we can see that Equation (47) applies also to multideterminantal wavefunctions, this time the index *i* changing from 1 to m(m+1)/2. Although, in the HF or single-determinant case, each $\lambda_{i}=-2$ for i=1,3,6,… and $\lambda_{i}=-4$ for the other *i*’s, in the multideterminantal case this is not so, and the deviations of $\lambda_{i}$ from the above values gives a measure of the multiconfigurational character of the wavefunction. In a similar way, each $G_{i}$ is the product of two canonical orbitals in the HF case, but it is a linear combination of orbital products in the general case.

#### 3.2.2. Coupled Cluster (Cc) Densities

Except in the case of a full-CI calculation, the coupled-cluster (CC) electronic structure method probably provides the most accurate results of quantum chemistry [25] at this moment. Contrarily to the HF approximation, that does not include dynamical correlation (DC) at all, or with CAS self-consistent-field (CASSCF) calculations, where most part of the included electron correlation is non dynamic, the CC theory including single and double excitations (CCSD) and, sometimes, third order excitations in a perturbative way (CCSD(T)), is able to recover a large percentage of the dynamic correlation energy. A first proposal to divide the Coupled Cluster energy according to the IQA partition was made by Rocha-Rinza and coworkers [37]. They use HF/CC transition densities to carry out such division. Unfortunately, that approach had the inconvenience of not being consistent with the calculation of other properties beyond the electronic energy. Later on, the same group put forward an implementation of the IQA energy partition based on CC Lagrangian one- and two-electron density matrices [38]. In this approach, the $D_{pq}$ and $d_{pqrs}$ coefficients of $\rho_{1}$ (Equation (42)) and $\rho_{2}$ (Equation (43)) are given as

(52) $$\begin{array}{ccc} D_{pq}^{\Lambda } & = & \langle \Lambda |{\hat{E}}_{pq}|\mathrm{CC}\rangle , \end{array}$$

(53) $$\begin{array}{ccc} d_{pqrs}^{\Lambda } & = & \langle \Lambda |{\hat{e}}_{pqrs}|\mathrm{CC}\rangle , \end{array}$$

where ${\hat{E}}_{pq}=\sum_{\sigma =\alpha ,\beta }{\hat{a}}_{p\sigma }^{†}{\hat{a}}_{q\sigma }$, ${\hat{a}}_{p\sigma }^{†}$ is a creation operator, ${\hat{a}}_{q\sigma }$ an annihilation operator, ${\hat{e}}_{pqrs}={\hat{E}}_{pq}{\hat{E}}_{rs}-\delta_{rq}{\hat{E}}_{ps}$,

(54) $$\langle \Lambda |=\langle \mathrm{HF}|+\sum_{\mu }{\bar{t}}_{\mu }\langle \mu |e^{-\hat{T}},$$

and ${\bar{t}}_{\mu }$ and 〈μ| are the Lagrange multipliers and the projection manifold of the CC equations, respectively. After substituting Equation (54) into Equations (52) and (53), one obtains the expressions

(55) $$\begin{array}{ccc} \;\;\;D_{pq}^{\Lambda } & \;\;=\;\; & \langle \mathrm{HF}|{\hat{E}}_{pq}|\mathrm{CC}\rangle +\sum_{\mu }{\bar{t}}_{\mu }\langle \mu |e^{-\hat{T}}{\hat{E}}_{pq}e^{\hat{T}}|\mathrm{HF}\rangle , \end{array}$$

(56) $$\begin{array}{ccc} \;\;\;d_{pqrs}^{\Lambda } & \;\;=\;\; & \langle \mathrm{HF}|{\hat{E}}_{pq}{\hat{E}}_{rs}|\mathrm{CC}\rangle \\ & + & \sum_{\mu }{\bar{t}}_{\mu }\langle \mu |e^{-\hat{T}}{\hat{E}}_{pq}{\hat{E}}_{rs}e^{\hat{T}}|\mathrm{HF}\rangle -\delta_{qr}D_{ps}^{\Lambda }. \end{array}$$

A detailed derivation of $D_{pq}^{\Lambda }$ and $d_{pqrs}^{\Lambda }$ will not be given here. Their final expressions for the CC single and double (CCSD) densities in terms of the single- ($t_{i}^{a}$) and double-excitation ($t_{ij}^{ab}$) CC amplitudes and the Lagrange multipliers ${\bar{t}}_{\mu }$ can be found in References [38,39].

After the CCSD coefficients $D_{pq}^{\Lambda }$ and $d_{pqrs}^{\Lambda }$ have been obtained, they are treated in the same way as in the case of multideterminantal wavefunctions: *D* is diagonalised to obtain the CCSD *natural* orbitals $\varphi_{i}$ and their occupation numbers $n_{i}$, and the η matrix defined in Equation (49), obtained from the $d_{pqrs}^{\Lambda }$ coefficients after subtracting the Coulomb part $\rho_{2}^{J}\left(r_{1},r_{2}\right)$ (Equation (12)) from the full two-electron density $\rho_{2}(r_{1},r_{2}$), diagonalised to obtain the eigenvalues $\lambda_{i}$ and eigenfunctions $G_{i}\left(r\right)$.

#### 3.2.3. Møller-Plesset Densities (Mpn)

The IQA partition combined with the MP2 approximation was successfully used for the first time by Popelier and coworkers to study electron correlation [40,41]. The problem with this initial IQA/MP2 approach is that its computational cost is practically the same as the one corresponding to an IQA/CCSD partition. However, a new algorithm has been recently developed that takes only into account occupied to virtual excitations [42]. The new IQA/MP2 method allows for dramatic reductions in computer time, although it has the disadvantage of providing first order properties similar to those of the HF approach. This is so because Møller-Plesset perturbation theory adds exclusively correlation energy corrections on top of the HF reference, $E\left(\mathrm{MP}\right)=E\left(\mathrm{HF}\right)+E_{\mathrm{corr}}$. Partitioning $E_{\mathrm{corr}}$ à la IQA leads to MP IQA correlation contributions. Restricting to second-order corrections (MP2) (although the treatment is easily generalised to the general MPn case), $E_{\mathrm{corr}}^{\mathrm{MP}2}$ for a closed-shell molecule is given by

(57) $$E_{\mathrm{corr}}^{\mathrm{MP}2}=\frac{1}{2}\sum_{iajb}\frac{2(2g_{iajb}-g_{ibja})}{\epsilon_{i}+\epsilon_{j}-\epsilon_{a}-\epsilon_{b}}g_{iajb},$$

where $g_{iajb}=(\phi_{i}\left(r_{1}\right)\phi_{a}^{⋆}\left(r_{1}\right)\left|r_{12}^{-1}\right|\phi_{j}\left(r_{2}\right)\phi_{b}^{⋆}\left(r_{2}\right)$ is a two-electron integral over canonical HF orbitals $\phi_{p}\left(r\right)$ with energy $\epsilon_{p}$, *p*, *q*, …, are general orbitals, and *i*, *j*, …, and *a*, *b*, …, run over occupied and virtual orbitals, respectively. The definition of the the effective correlation matrix elements $d_{iajb}=2\left(2g_{iajb}-g_{ibja}\right)/\left(\epsilon_{i}+\epsilon_{j}-\epsilon_{a}-\epsilon_{b}\right)$ allows to define an effective second-order $\rho_{2}^{\mathrm{eff}}$ density as

(58) $$\rho_{2}^{\mathrm{eff}}\left(r_{1},r_{2}\right)=\rho_{2}^{\mathrm{HF}}\left(r_{1},r_{2}\right)+\rho_{2}^{\mathrm{MP}2}\left(r_{1},r_{2}\right),$$

where

(59) $$\rho_{2}^{\mathrm{MP}2}\left(r_{1},r_{2}\right)=\sum_{iajb}d_{iajb}\phi_{i}\left(r_{1}\right)\phi_{a}^{⋆}\left(r_{1}\right)\phi_{j}\left(r_{2}\right)\phi_{b}^{⋆}\left(r_{2}\right).$$

Since matrix elements iijj or ijij are absent from $\rho_{2}^{\mathrm{MP}2}$, $\rho_{2}^{\mathrm{eff}}$ integrates to the HF density,

(60) $$\int \rho_{2}^{\mathrm{eff}}\left(r_{1},r_{2};r_{1}^{\prime },r_{2}\right)dr_{2}=\left(N-1\right)\rho^{\mathrm{HF}}\left(r_{1};r_{1}^{\prime }\right),$$

so that all one-electron properties and standard QTAIM descriptors remain unaltered and equal to their HF values. Similarly, since the total 2RDM is the sum of its HF component plus the MP2 term (Equation (58)), all Coulomb and exchange energies are also unchanged with respect to their HF counterparts, and therefore

(61) $$\begin{array}{ccc} E_{\mathrm{corr}}^{\mathrm{MP}2} & = & \frac{1}{2}\sum_{iajb}d_{iajb}g_{iajb}, \end{array}$$

(62) $$\begin{array}{ccc} & = & \sum_{A}E_{\mathrm{corr},\mathrm{MP}2}^{A}+\sum_{A>B}E_{\mathrm{corr},\mathrm{MP}2}^{AB}. \end{array}$$

This is the partition of the total MP2 correlation energy into intra-atomic ($E_{\mathrm{corr},\mathrm{MP}2}^{A}$) and inter-atomic ($E_{\mathrm{corr},\mathrm{MP}2}^{AB}$) contributions. The structure of Equation (61) allows a considerable saving of effort. The η array (Equation (51)) that has to be diagonalised in the CASSCF, Full-CI, or CCSD IQA implementations has a size of m(m+1)/2, where m=o+v is the total number of occupied) (*o*) plus virtual (*v*) orbitals in the basis. In the MP2 case, however, due to Equation (58), η has the structure depicted in Table 1, where empty (○) and filled (●) circles represent zero and non-zero values, respectively.

The oo block, containing the HF exchange energy, is already diagonal, the vv block is zero, and the ov block, of dimension (ov×ov), is the only one that needs to be diagonalised. In typical MP2 calculations intended for real-life chemical problems, m>>o and v>>o, and the effort needed to numerically calculate the six-dimensional integrals involved in the computation of $E_{\mathrm{corr},\mathrm{MP}2}^{A}$ and $E_{\mathrm{corr},\mathrm{MP}2}^{AB}$ is reduced by a factor that scales as v/o. This computational saving allows the treatment of much larger systems in reasonable computed times.

#### 3.2.4. Kohn-Sham Densities (Dft)

There is no second order density within the Kohn-Sham (KS) approximation. Hence, the IQA method is not cleanly defined in DFT. However, given the increasing importance of DFT calculations in ever-expanding fields of Computational Chemistry, it was mandatory to carry out a formulation of IQA in a DFT context. Popelier et al. were the first to give a first implementation of the IQA/DFT partition within the KS formalism of DFT in conjunction with the B3LYP functional [43]. The same year, Francisco et al. presented another formulation that can be applied with a large number of hybrid and non-hybrid xc functionals [44]. The starting point of the latter is the comparison between the xc DFT energy ($E_{xc}^{\mathrm{DFT}}$) and the exchange-only (*x*) energy of a KS determinant, $E_{x}^{\mathrm{KS}}$, formally analogous to the xc energy of a single-determinant wavefunction, Equation (15). For a non-hybrid xc DFT functional ε(r)≡ε[ρ(r),|∇ρ(r)|] (the treatment of hybrid xc functionals is very similar to this one and is discussed in detail in Reference [44]) $E_{xc}^{\mathrm{DFT}}$ is given by

(63) $$E_{xc}^{\mathrm{DFT}}=\int dr\rho \left(r\right)\varepsilon \left(r\right).$$

On the other hand,

(64) $$E_{x}^{\mathrm{KS}}=\frac{1}{2}\;\int \int \;dr_{1}dr_{2}\frac{\rho_{2}^{x,\mathrm{KS}}\left(r_{1},r_{2}\right)}{r_{12}},$$

where $\rho_{2}^{x,\mathrm{KS}}\left(r_{1},r_{2}\right)$ is the *x* density of the KS determinant. After $R^{3}$ is partitioned into disjoint domains, $E_{xc}^{\mathrm{DFT}}$ and $E_{x}^{\mathrm{KS}}$ can be written in the form

(65) $$E_{xc}^{\mathrm{DFT}}=\sum_{A}\int_{A}\rho \left(r\right)\varepsilon \left(r\right)dr=\sum_{A}E_{xc}^{A,\mathrm{DFT}},\;\mathrm{and}$$

(66) $$\begin{array}{ccc} E_{x}^{\mathrm{KS}} & = & \sum_{A}E_{x}^{A,\mathrm{KS}},\;\mathrm{where} \end{array}$$

(67) $$\begin{array}{ccc} E_{x}^{A,\mathrm{KS}} & = & E_{x}^{AA,\mathrm{KS}}+\frac{1}{2}\sum_{B\neq A}E_{x}^{AB,\mathrm{KS}}, \end{array}$$

(68) $$\begin{array}{ccc} E_{x}^{AA,\mathrm{KS}} & = & \frac{1}{2}\int_{A}dr_{1}\int_{A}dr_{2}\;\frac{\rho_{2}^{x,\mathrm{KS}}\left(r_{1},r_{2}\right)}{r_{12}}, \end{array}$$

(69) $$\begin{array}{ccc} E_{x}^{AB,\mathrm{KS}} & = & \int_{A}dr_{1}\int_{B}dr_{2}\;\frac{\rho_{2}^{x,\mathrm{KS}}\left(r_{1},r_{2}\right)}{r_{12}}. \end{array}$$

The subscript *x* instead of xc in the above quantities emphasises that they do no recover the full xc energies of a DFT calculation. To accomplish this objective, we define the $\lambda_{A}$ quantity as

(70) $$\lambda_{A}=\frac{E_{xc}^{A,\mathrm{DFT}}}{E_{x}^{A,\mathrm{KS}}},$$

and define the scaled quantities ${\tilde{E}}_{xc}^{AA}$ and ${\tilde{E}}_{xc}^{AB}$ by

(71) $${\tilde{E}}_{xc}^{AB}=\frac{1}{2}\left[\lambda_{A}+\lambda_{B}\right]E_{x}^{AB,\mathrm{KS}},$$

both A=B and A≠B. It is then straightforward to show that

(72) $$\begin{array}{ccc} E_{xc}^{\mathrm{DFT}} & = & \sum_{A}{\tilde{E}}_{xc}^{\mathrm{AA}}+\sum_{A>B}{\tilde{E}}_{xc}^{AB} \end{array}$$

(73) $$\begin{array}{ccc} & = & \sum_{A}\left[{\tilde{E}}_{xc}^{AA}+\frac{1}{2}\sum_{B\neq A}{\tilde{E}}_{xc}^{AB}\right]=\sum_{A}{\tilde{E}}_{xc}^{A}. \end{array}$$

This is the desired partition of $E_{\mathrm{xc}}^{\mathrm{DFT}}$ in terms of intra-atomic (${\tilde{E}}_{\mathrm{xc}}^{\mathrm{AA}}$) and inter-atomic (${\tilde{E}}_{\mathrm{xc}}^{\mathrm{AB}}$) contributions. Equation (72) recovers exactly the full xc energy of the DFT calculation.

The above strategy to scale the intra- and inter-atomic xc energies such that the total DFT energy is exactly recovered differs from that used by the Aimall code [43], where this requirement is achieved by leaving unchanged the inter-atomic term, $E_{x}^{AB,\mathrm{KS}}$ (A≠B), which is evaluated using Equation (69), and modifying each intra-atomic term according to $E_{xc}^{AA}\left({}_{\mathrm{AIMALL}}\right)=E_{xc}^{A,\mathrm{DFT}}-\frac{1}{2}\sum_{B\neq A}E_{x}^{AB,\mathrm{KS}}$.

#### 3.2.5. Other Approximate Densities

Besides all the above $\rho_{2}$ and $\rho_{2}^{xc}$ densities, the IQA partition can also deal with approximate xc densities that do not require the knowledge of the molecular wavefunction, but only the natural orbitals $\varphi_{p}$ and their occupation numbers $n_{p}$ (Equation (48)) [45]. Many approximate forms for $\rho_{2}^{xc}$ have been proposed within the Density Matrix Functional Theory (DMFT) with the general formula [45]

(74) $$\rho_{2}^{\mathrm{xc}}\left(r_{1};r_{2}\right)=\sum_{pq}f\left(n_{p},n_{q}\right)\;\chi_{pq}\left(r_{1}\right)\;\chi_{pq}\left(r_{2}\right),$$

where $\chi_{pq}=\varphi_{p}\varphi_{q}$. Different $f(n_{p},n_{q})$’s provide different approximate $\rho_{2}^{xc}$’s. The most widely used and well-known proposal is the so-called Müller or Buijse-Baerends functional (BB) that, in closed-shell systems, takes the form [46,47] $f^{\mathrm{BB}}\left(n_{i},n_{j}\right)=2{\left(n_{i}n_{j}\right)}^{\frac{1}{2}}$.

Other improved possibilities, which we will not discuss here for brevity, are those due to Goedecker and Umrigar [48] (GU), Csányi and Arias (CA) [49], Csányi, Goedecker and Arias [50], the hybrid (GU+CA) functional proposed by Staroverov and Scuseria [51], and the corrected BB expressions given by Gritchenko et al. [52] in an attempt to correct the overcorrelation of the Müller functional while preserving the proper description in the dissociation limit, and so forth. Finally, the successive improved formulas of Piris and coworkers also deserve special mention [53]. All these possibilities share the common feature of providing diagonal expressions for $\rho_{2}^{xc}$ in the basis of products of natural MOs. Therefore, the diagonalisation of η matrix (see Equation (51)) is not required in any of the above cases.

### 3.3. Practical Aspects of Iqa Implementation

The most expensive computational task of any IQA implementation lies in the numerical evaluation of the one-electron tridimensional and two-electron hexadimensional integrals over the, generally, irregular domains $\Omega_{A}$ in which QTAIM divides the physical space. We will not discuss in this review how different implementations of the IQA method deal with these integrations. As regards monoelectronic integrations, the literature on the subject is quite extensive. Even the description of the many methods that have been proposed so far to integrate ρ(r) and different functions of it in the QTAIM $\Omega_{A}$ regions would require a review in its own. On the contrary, we will settle for discussing in detail, although not exhaustively, the algorithms developed in our group and incorporated in the domestic program Promolden. This is not the most popular implementation of IQA. This privilege corresponds to the one included in the Aimall code. However, as far as we are aware of, Aimall cannot deal yet with the large variety of second order densities that Promolden can handle almost as easily as if they were Hartree-Fock densities.

We will consider separately the integration of monoelectronic and bielectronic IQA energies. Although some elements of both integrations are common, they differ quite a bit in other respects, so it is appropriate to differentiate both treatments. In any case, one element which is common to all the integrations made within the Promolden program is the interatomic surface $S(\Omega_{A})$, that separates a QTAIM domain $\Omega_{A}$ from other atomic basins. Centring a spherical coordinate system in the nucleus of *A*, we will call $R(\hat{r})$, with $\hat{r}\equiv \left(\theta ,\phi \right)$, the maximum distance from this nucleus to $S(\Omega_{A})$. In some cases $R(\hat{r})\to \infty$. When this happens, Promolden takes $R\left(\hat{r}\right)=R_{\max}$, where $R_{\max}$ is large enough to ensure that the density for larger values is completely negligible. Different $R_{\max}$’s can be chosen for the different atoms of the system. In other case, it may happen that a line leaving the nucleus of *A* with the direction $\hat{r}$ cuts $S(\Omega_{A})$ at various points. This event is explicitly taken into account by Promolden. In other words, Promolden always foresees the possibility of multiple intersections with $S(\Omega_{A})$. The latter is determined in the program by means of a bipartition algorithm that, although it may not be extremely fast, is quite robust.

#### 3.3.1. Integration Schemes: Monoelectronic Terms

There are three different forms in which monoelectronic energy components can be integrated in Promolden. Let

(75) $$I=\int_{\Omega }f\left(r\right)\;dr,$$

where f(r) is the integrator of any of the monoelectronic energy terms previously seen. In the first method (standard method), we define $f^{\Omega }\left(r\right)=f\left(r\right)\;\omega_{\Omega }\left(r\right)$ and transform Equation (75) to

(76) $$I=\int f^{\Omega }\left(r\right)\;dr,$$

that is, the integration of *f* within the basin Ω is replaced by the integration of $f^{\Omega }\left(r\right)$ in the whole space. Now, the angular integration of $f^{\Omega }\left(r\right)$ over the angles $\left(\theta ,\phi \right)\equiv \hat{r}$ is performed, obtaining the radial function $f^{\Omega }\left(r\right)$

(77) $$f^{\Omega }\left(r\right)=\int_{\hat{r}}f^{\Omega }\left(r\right)\;d\hat{r},$$

($d\hat{r}=\sin\left(\theta \right)d\theta d\phi$) and, finally, integrate $f^{\Omega }\left(r\right)$ over the radial coordinate *r*:

(78) $$I=\int_{r=0}^{R_{\max}}r^{2}\;f^{\Omega }\left(r\right)\;dr.$$

Several quadratures can be used to obtain $f^{\Omega }\left(r\right)$, although a Lebedev angular grid [54,55] with a variable number of points, is used most times. To compute the radial integral in Equation (78), the interval $r\in [0,R_{\max}]$ is mapped onto a new finite interval u∈[−1,+1] (or u∈[0,+1]) by means of a coordinate transformation r(u) such that r(−1)=0 (or r(0)=0) and $r\left(+1\right)=R_{\max}$. Several r(u) possibilities are included in Promolden.

Using the Heaviside-like function $\omega_{\Omega }\left(r\right)$ in the definition of $f^{\Omega }\left(r\right)$ causes sharp jumps in the values of this function when $\hat{r}$ is changed for a given value of *r* when performing integration (77). This forces the use of angular grids with a considerably high number of $\hat{r}$ points. However, as a counterpart, performing the angular integration for each *r* value instead of the radial integration for each $\hat{r}$, and not the other way around, is what gives bi-electronic integrations a very favourable scaling. It is implicit in Equation (78) that the present integration scheme requires a single set of radial grid points for each atomic basin Ω.

In the second monoelectronic integration scheme included in Promolden, the order of the radial and angular integrations is reversed. For each angular grid point $\hat{r}$, the integral

(79) $$I\left(\hat{r}\right)=\int_{r=0}^{R(\hat{r})}r^{2}\;f\left(r\right)\;dr$$

is computed first. Then, the angular integration is performed

(80) $$I=\int_{\hat{r}}I\left(\hat{r}\right)\;d\hat{r}.$$

This method requires a different radial grid for each $\hat{r}$ point of every monocentric integration, which adds a negligible CPU time to the total necessary to perform a typical IQA calculation.

Finally, the last monoelectronic integration scheme included in Promolden is a variant of the above, in which all radial integrations are obtained using the QUADPACK library [56]. Each integral is obtained with a precision greater than an absolute error predefined in advance by successively dividing the radial interval up to a maximum number of times that is also previously defined.

#### 3.3.2. Integration Schemes: Bielectronic Terms

A first point that we must highlight here is that, as it can be inferred from Section 3.2, all the bielectronic integrals that appear in the present implementation of the IQA method have the form

(81) $$I^{AB}=\int_{A}dr_{1}\int_{B}dr_{2}\;\frac{f\left(r_{1}\right)\;f\left(r_{2}\right)}{r_{12}},$$

with both monoelectronic functions being the same, f(r), and where *B* can be equal to (monocentric) or different from *A*. If $I^{AB}$ refers to a classical electron-electron interaction, f(r)=ρ(r), while in exchange-correlation interactions f(r)=G(r), where G(r) is the product of two canonical or Kohn-Sham orbitals (in HF wavefunctions and Kohn-Sham *pseudo-*wavefunctions, respectively) or a linear combination of these products in correlated descriptions (CASSCF, CCSD, etc.). The fact that the same function is evaluated with the arguments $r_{1}$ and $r_{2}$ is what confers this IQA implementation its very favourable scaling properties with the increase of the size of the system [22].

**Figure 1:**
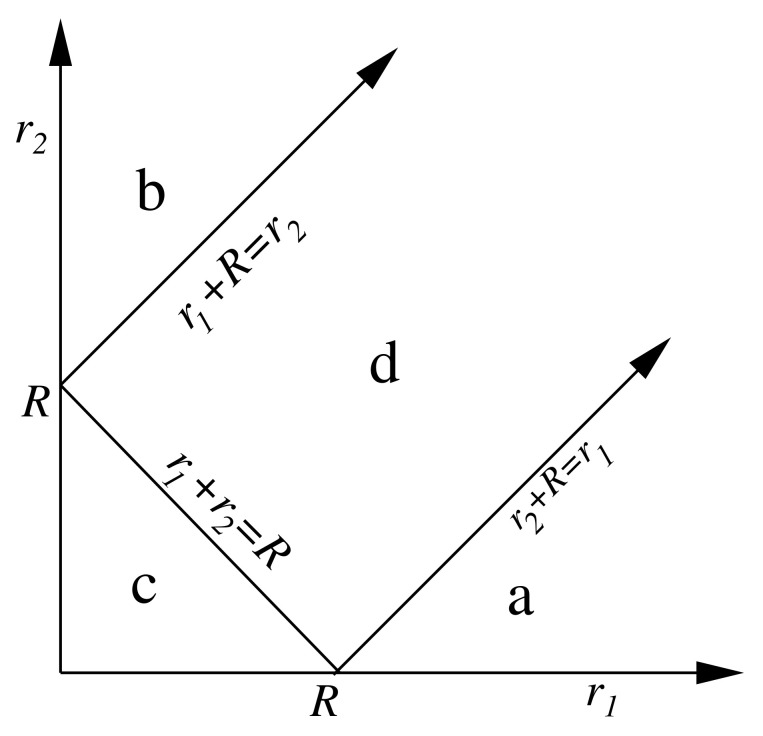
Regions of definition of the $D_{l_{1}m_{1}}^{l_{2}m_{2}}\left(r_{1},r_{2},R\right)$ function.

To advance in the calculation of $I^{AB}$ it is necessary to disentangle the $r_{1}$ and $r_{2}$ coordinates in Equation (81). Full details of how this disentanglement is carried out appear in the original reference [34]. Here, only a summary of this process is given. The treatment is rather different for the monocentric ($I^{AA}$) and bicentric $I^{AB}$ (A≠B) integrals. In the first case, the well-known Laplace expansion for $r_{12}^{-1}$ is used:

(82) $$r_{12}^{-1}=\sum_{l=0}^{\;\infty }\frac{4\pi }{2l+1}\frac{r_{<}^{l}}{r_{>}^{l+1}}\sum_{m=-l}^{+l}S_{lm}\left({\hat{r}}_{1}\right)S_{lm}\left({\hat{r}}_{2}\right),$$

where $S_{lm}\left(\hat{r}\right)$ is a real spherical harmonic [57], and $r_{<}$ and $r_{>}$ correspond to the smaller and the larger of $\left(r_{1},r_{2}\right)$. In the bicentric case (A≠B), the less-known bipolar expansion for $r_{12}^{-1}$ is used [58,59]:

(83) $$r_{12}^{-1}=\sum_{l_{1}m_{1}}^{\;\infty }\sum_{l_{2}m_{2}}^{\;\infty }S_{l_{1}m_{1}}\left({\hat{r}}_{1}\right)\;S_{l_{2}m_{2}}\left({\hat{r}}_{2}\right)\;D_{l_{1}m_{1}}^{l_{2}m_{2}}\left(r_{1},r_{2},r\right),$$

where $r_{1}\equiv \left(r_{1},{\hat{r}}_{1}\right)$ and $r_{2}\equiv \left(r_{2},{\hat{r}}_{2}\right)$ are referred to centres *A* and *B*, respectively, $R=\left(R_{B}-R_{A}\right)\equiv \left(R,\hat{R}\right)$ is the vector position of center *B* with respect to center *A*, and $D_{l_{1}m_{1}}^{l_{2}m_{2}}\left(r_{1},r_{2},R\right)$ is a function defined in a different way in the four regions *a*, *b*, *c*, and *d* depicted in Figure 1.

To carry out the integration over $r_{1}$ and $r_{2}$ in Equation (81) we use again the method 1 discussed in monoelectronic integrations. Using Equation (82) in Equation (81) with A=B, we have

(84) $$\begin{array}{ccc} I^{AA} & = & \sum_{lm}^{\;\infty }\;\int \int \;\left(\frac{r_{<}^{l}}{r_{>}^{l+1}}\right)\;f_{lm}^{\Omega_{A}}\left(r_{1}\right)\;f_{lm}^{\Omega_{A}}\left(r_{2}\right)\;r_{1}^{2}\;r_{2}^{2}\;dr_{1}\;dr_{2}=\sum_{lm}^{\;\infty }I_{lm}^{AA}, \end{array}$$

where $f_{lm}^{\Omega }\left(r\right)=N_{l}\;\int_{\hat{r}}S_{lm}\left(\hat{r}\right)\;f^{\Omega }\left(r\right)\;d\hat{r}$ and $N_{l}=\sqrt{4\pi /(2l+1)}$. As in the monoelectronic integration, the ∞ upper limit of $r_{1}$ and $r_{2}$ in Equation (84) is replaced by a finite value $R_{\max}$ which guarantees that $f_{lm}^{\Omega }\left(r\right)≃0$ for $r>R_{\max}$, and map the $\left[0,R_{\max}\right]$ interval onto a new finite interval u∈[−1,+1] or u∈[0,+1] by using a $r_{1}\left(u_{1}\right)$ and $r_{2}\left(u_{2}\right)$ coordinate transformation. Using $N_{r}+1$ points $u_{i}$ and weights $w_{i}$ ($i=0,1,\ldots ,N_{r}$), we can write $I_{lm}^{AA}$ as

(85) $$I_{lm}^{AA}\;\;=\;\;\sum_{i,j=0}^{N_{r}}\frac{r_{<}^{l}}{r_{>}^{l+1}}\;f_{lm}^{\Omega_{A}}\left(r_{i}\right)\;f_{lm}^{\Omega_{A}}\left(r_{j}\right)\;r_{i}^{2}\;r_{j}^{2}\;r^{\prime }\left(u_{i}\right)\;r^{\prime }\left(u_{j}\right)\;w_{i}\;w_{j},$$

where $r_{i}=r\left(u_{i}\right)$ and $r^{\prime }\left(u\right)=\left(dr/du\right)$. A single $f_{lm}^{\Omega }\left(r\right)$ function suffices to determine $I_{lm}^{AA}$ through a double radial integration. As described in Reference [34], this fact can be used to further reduce the complexity of the intra-atomic integration.

To compute the bielectronic inter-atomic integrals $I^{AB}$ we replace expression (83) for $r_{12}^{-1}$ in Equation (81) (A≠B) to give

(86) $$I^{AB}=\sum_{l_{1}m_{1}}^{\infty }\sum_{l_{2}m_{2}}^{\infty }I_{l_{1}m_{1},l_{2}m_{2}}^{AB},\;\mathrm{with}$$

(87) $$\begin{array}{ccc} I_{l_{1}m_{1},l_{2}m_{2}}^{AB} & = & N_{l_{1}}^{-1}N_{l_{2}}^{-1}\times \int f_{l_{1}m_{1}}^{\Omega_{A}}\left(r_{1}\right)\;r_{1}^{2}\;dr_{1}\times \int f_{l_{2}m_{2}}^{\Omega_{B}}\left(r_{2}\right)\;r_{2}^{2}\;dr_{2}\;D_{l_{1}m_{1}}^{l_{2}m_{2}}\left(r_{1},r_{2},R\right). \end{array}$$

Since the two $f_{lm}^{\Omega }\left(r\right)$ can be independently computed, the original problem, with a computational complexity that scales as $N^{6}$, has been transformed into a more tractable $2N^{4}$ problem. This allows for great savings in computer time, which can be even larger by precomputing and reusing the $f_{lm}^{\Omega }\left(r\right)$ functions in different integrals involving the same atom Ω, for both intra- and inter-atomic integrals.

It becomes clear from Figure 1 that $I^{AB}$, and also $I_{l_{1}m_{1},l_{2}m_{2}}^{AB}$, is a sum of four contributions,

(88) $$I^{AB}=I_{a}^{AB}+I_{b}^{AB}+I_{c}^{AB}+I_{c}^{AB},$$

corresponding to the four different functional forms that the $D_{l_{1}m_{1}}^{l_{2}m_{2}}\left(r_{1},r_{2},r\right)$ function has in the four regions *a*, *b*, *c*, and *d* in which the $\left(r_{1},r_{2}\right)$ plane has been divided [60]. Despite this, $D_{l_{1}m_{1}}^{l_{2}m_{2}}\left(r_{1},r_{2},r\right)$ is continuous and differentiable for any $r_{1}$ and $r_{2}$ values, so that each $I_{l_{1}m_{1},l_{2}m_{2}}^{AB}$ can be computed by a double mapped-radial quadrature similar to that used for $I_{lm}^{AA}$, previously replacing the ∞ upper limit of $r_{1}$ and $r_{2}$ by $R_{\max}^{A}$ and $R_{\max}^{B}$, respectively.

The *l* expansion in the expression of the monocentric energy terms, $I^{AA}$ (Equation (84)), is carried out up to a maximum value $l=l_{\max}$ defined in advance, while the *l* expansion in the bicentric terms (Equation (86)) is performed up to $l_{\max}$ or until the sum has converged with a given precision, whichever comes first.

#### 3.3.3. The Multipolar Approach for the Classical and Exchange-Correlation Energies

An important simplification in the calculation of $I^{AB}$ occurs when regions A and B are widely separated in space. As long as the radial coordinates $r_{1}$ and $r_{2}$ in Equation (87) satisfy $r_{1}+r_{2}\leq R$, the function $D_{l_{1}m_{1}}^{l_{2}m_{2}}\left(r_{1},r_{2},r\right)$ has to be evaluated only in region *c* of Figure 1. This strictly happens only when the sum of the maximum values of $r_{1}$ and $r_{2}$, $R_{\max}^{A}+R_{\max}^{A}$, is less than or equal to the distance between the nuclei of regions *A* and *B*, that is, $R_{\max}^{A}+R_{\max}^{A}<R$. This condition is what defines the non-overlapping character of two regions in IQA bicentric integration, and differs from that commonly used, in which two regions of $R^{3}$ are said to be overlapping if the intersection between them is a non-null region of this space.

The multipolar approximation, widely used to compute the classical interatomic interaction in the modellisation of biomolecules, is equivalent to assume that the above IQA non-overlapping condition is always satisfied. In other words, region c of Figure 1 is identified with the complete first quadrant. After a lenghty but easy manipulation, that we omit here for brevity, we get

(89) $$I^{AB}≃\sum_{l_{1}m_{1}}^{\;\infty }\sum_{l_{2}m_{2}}^{\;\infty }C_{l_{1}m_{1}}^{l_{2}m_{2}}\left(\hat{R}\right)\frac{f_{l_{1}m_{1}}^{\Omega_{A}}f_{l_{2}m_{2}}^{\Omega_{B}}}{R^{l_{1}+l_{2}+1}},$$

where $C_{l_{1}m_{1}}^{l_{2}m_{2}}\left(\hat{R}\right)$ are angular coefficients [34], and $f_{lm}^{\Omega }=N_{l}\int_{\Omega }r^{l}\;S_{lm}\left(\hat{r}\right)f\left(r\right)dr$. In case that f(r)=ρ(r), $f_{lm}^{\Omega }$ are the spherical multipoles $Q_{lm}^{\Omega }$ of standard classical multipolar interactions [3]: $Q_{00}^{\Omega }$ is the total electronic charge in Ω, $Q_{1m}^{\Omega }$ (m=+1,−1,0) the Cartesian dipoles of Ω ($\mu_{x}^{\Omega },\mu_{y}^{\Omega },\mu_{z}^{\Omega }$), and so on.

The multipolar approximation can be applied to compute, not only the classical Coulomb interatomic energy, but also the exchange-correlation bicentric terms. Using it can save huge amounts of CPU time. Our domestic code Promolden optionally includes that possibility [61]. However, by default, all bicentric interactions are always calculated using the exact expression for $D_{l_{1}m_{1}}^{l_{2}m_{2}}$, that is, the one corresponding to given values of $r_{1}$ and $r_{2}$. Doing it this way, the expression 87 is always absolutely convergent, regardless the values of $l_{1}m_{1}$ and $l_{2}m_{2}$. Moreover, even if the multipolar approach converges to a defined value of a given interaction, there is not guarantee that this value coincides the exact one.

#### 3.3.4. Increasing the Precision of Iqa Integrations: Atomic β-Spheres

The irregular shapes of QTAIM atomic domains $\Omega_{A}$ are a challenge for numerical integration quadratures. Sometimes, even with a very large number of radial ($N_{r}$) and angular ($N_{\theta ,\phi }$) points, it is impossible to achieve a good convergence in the integrations. A test of the quality of the latter is the total energy of the molecule, that ideally should be equal to that obtained in the electronic structure calculation performed to obtain the wavefunction of the system, or the total charge of the molecule, which is known beforehand. The usual way of trying to improve this convergence, both in the original QTAIM integrations of ρ(r) as in the IQA method, is the use of atomic β-spheres [54]. Each atomic basin $\Omega_{A}$ is divided into two-subregions, $\Omega_{A}=\beta_{A}+\gamma_{A}$ ($\beta_{A}\equiv \beta$-sphere region of $\Omega_{A}$,$\gamma_{A}\equiv$ no-β-sphere region of $\Omega_{A}$). Then, each monoelectronic integral (Equation (75)) is the sum of two contributions, I=I(β)+I(γ), and each bielectronic integral $I^{AB}$ (A=B or A≠B, Equation (81)) the sum of four terms

(90) $$I^{AB}=I_{\beta \beta }^{AB}+I_{\gamma \beta }^{AB}+I_{\beta \gamma }^{AB}+I_{\gamma \gamma }^{AB}.$$

The β-spheres radii ($R_{\beta }$) can be freely chosen, with the only requirement that they do not cut the interatomic surface S(Ω). For this, $R_{\beta }$ must be less than the shortest distance from the atomic nucleus to S(Ω). Any two β-sphere regions $\beta_{A}$ and $\beta_{B}$ are, by definition, non-overlapping in the sense discussed above. Hence, the $I_{\beta \beta }^{AB}$ term is given exactly by the expression Equation (89), provided $f_{lm}^{\Omega }$ are replaced by the β-sphere spherical multipoles, $f_{lm}^{\beta }$.

It might appear at first glance that the greater the value $R_{\beta }$ that meets the non-overlapping domains condition and, consequently, the greater the region of the $\left(r_{1},r_{2}\right)$ quadrant for which it is possible to apply the multipolar approximation, the more precise would be the calculated value of a given integral $I^{AB}$. While this is so in many cases, in others it is not. In practice, it seems that $R_{\beta }$ values around 40–60% the minimum distance from the atomic nucleus to S(Ω) is a close-to-optimal choice in most cases.

## 4. Selected Applications of the Iqa Methodology

IQA has been successfully employed in the study of a wide variety of chemical problems. Herein we present a selection of this applications. We tried to give an accurate overview of the diverse work carried out by a variety of research groups around the globe.

As it is natural, the first applications of IQA were on intramolecular interactions in systems of moderate size. For instance, Martín Pendás et al. produced a seminal work regarding the use of IQA in the study bonding properties using diatomic molecules of the first row as examples [62]. Later on, more research has emerged regarding the use of IQA to investigate intramolecular chemical interactions. For example, García-Revilla and coworkers studied the (O${}_{2}$)${}_{4}$ [63] cluster while Belyacob et al. investigated. gaseous proline [64]. Moreover, Bartashevich and coworkers [65] and Mitzel et al. [66] have studied the internal contacts in halogen-substituted trinitromethanes, or Cukrowski and Mangondo who investigated the interactions in beryllium complexes with nitrilotriacetic and nitrilotri-3-propionic acids [67].

Converserly, there have been a strong push for approximations that would allow for the application of IQA on larger, more complex systems. For instance, the kernel energy method, a fragmentation approximation intended to calculate different properties of large molecules relatively quickly and accurately, has been applied to atomic energies with mostly favorable results [68].

### 4.1. Electronic Correlation

The study of electronic correlation has also benefited by the application of IQA. A first effort in this direction was carried out by Rocha-Rinza et al. who studied separately Fermi and Coulomb correlation [69]. Popelier et al. analysed the effects of electron correlation within atoms and between atoms, bonded or not, under the MP2 approximation [70] and later on examined how higher orders of perturbation theory affect electronic correlation [71]. More recently, the same authors compared intra- and interatomic electron correlation energies resulting from of MP4SDQ and CCSD calculations finding that the later produces correlation energies that are much larger in magnitude [72]. Casals-Sainz used IQA in combination with CCSD(T) coupled cluster densities to investigate the spatial distribution of electronic correlation. They show that the interatomic electronic at long-range can be identified with dispersion [73].

### 4.2. Relationships between V${}_{\mathrm{xc}}$ and Di

From theory, the linear connection to the delocalization index between the covalent energy associated with an interaction, V${}_{\mathrm{xc}}$, and the corresponding delocalisation index, DI, is well known. Jara-Cortés and Hernández-Trujillo investigated further this relationship in aromatic, antiaromatic, and nonaromatic organic molecules and confirmed these regularities [74]. Badria and Foroutan-Nejad working exclusively on aromatic compounds found only a marginal deviation from the ideal linear behaviour of DI with respect to V${}_{\mathrm{xc}}$ [75].

Martín Pendás and Francisco extended this idea of this connection to include the so-called ionic bond order, an analogous of DI, by expanding the covalent and ionic interaction energies as multipolar series. They shown how the terms dominating both energies are the zeroth order ones, and thus correspond to distance-scaled bond orders [76].

### 4.3. The Nature of Chemical Bonding

**Figure 2:**
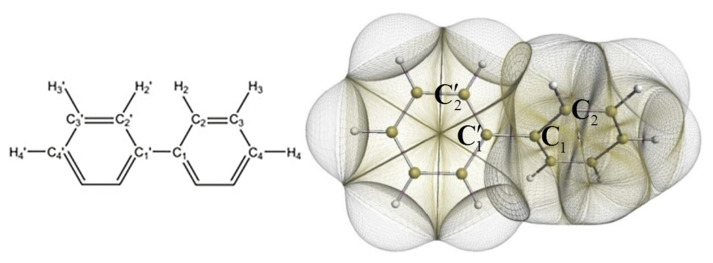
Biphenyl representations. Schematic (**left**) and non-overlapping topological atoms bounded by interatomic surfaces (**right**). Figure from reference [80].

IQA has found application in the investigation of chemical bonding in cases where the nature of the interaction between atoms is uncertain, in particular regarding the relationship between QTAIM bond paths bonds. For example, IQA has been used to study hydrogen–hydrogen bonding in planar biphenyl (represented in Figure 2), a controversial subject in the literature. For instance, studies applying QTAIM to this problem have found a bond path between H atoms and declared it as evidence of attractive interaction [77,78]. It has been proposed by Martín Pendás and coworkers that bond paths are preferred exchange channels its occurrence are independent from electrostatics factors or changes in the self energies of the involved atoms [29]. Eskandari and Van Alsenoy found the H–H contact attractive, although this contribution is cancelled out by very destabilising changes in the intraatomic energies [79]. Popelier et al. also studied the biphenyl outside the equilibrium and coincide in saying that the H–H contact in the planar configuration should not be considered as repulsive [80]. Additionally, other research groups have studied the H–H contact in alternative systems reaching similar conclusions; Mallia et al. investigated the intramolecular C-H…H-C interactions in the crystal of triphenylamine substituted arenes finding these interactions by themselves can be considered attractive [81]. Matczak showed that C-H...H-C contacts diheteroaryl ketones and thioketones conduct to a energetic total destabilisation in spite of the interaction between H atoms being weakly attractive [82].

Additionally, IQA has been used to discern the nature of the intramolecular interactions in situations where the energetic relationship between atoms or functional groups is controversial. Demyanov and Polestshuk studied the nature of interactions between host and guest in the HeAdamantane complex, establishing that there energy of the He–C contact is repulsive [83] while Tognetti and Joubert shown that the intramolecular interactions between electronegative atoms can also be attractive [84]. More recently, Dem’yanov and Polestshuk studied small ionic complexes and their work strongly suggest an altogether disconnection between bond paths and bonding interactions [85].

### 4.4. Non-Covalent Interactions

The modelling of non-covalent interactions is usually a complex matter given the relatively low but important energy associated. Foroutan-Nejad et al. utilised IQA to explore the nature of the anion-π bonding. Contrary to the classical vision of these contacts as mostly electrostatic, they found that the covalent contribution plays a central role in the stability of these complexes. They use this information to suggest that the recipe to prepare stable anion-π complexes is to use more extended π-systems rather than strong electrostatic π-receptors [86]. These authors also investigated lone pair-π interactions, finding, as in the previous case, that the exchange-correlation contribution to the interaction energy is relatively large. However, for the lone pair-π systems, this contribution is almost completely counterbalanced by the deformation energy. Thus, the determining factor for the formation of these types of contacts lies in the electrostatics of the fragments [87].

In a systematic effort for validating the use of IQA energies for the study of non-covalent interactions, Suárez et al. analysed the binding energies, magnitude of numerical errors, and the fragment and atomic distribution of formation energies within the IQA framework for the S66 and ionic-hydrogen-bond data sets. They shown that this energy partition rigorously quantify atomic and group energy contributions for biomolecular systems [88].

Non-covalent interactions involving larger systems have also been studied using IQA. Mitoraj and coworkers used the IQA methodology to quantify the different non-covalent interactions present in the LiN(CH${}_{3}$)${}_{2}$BH${}_{3}$ and KN(CH${}_{3}$)${}_{2}$BH${}_{3}$ crystals, commonly utilised as hydrogen storage materials [89]. Yourdkhani et al. studied the adsorption of a series organophosphor molecules by graphene [90].

#### 4.4.1. Hydrogen Bonding

Among the first applications of the IQA methodology was the study of the hydrogen bond by means of the examination of small dimers. The emerging picture from this seminal work of HB is one of an interaction characterised by both electrostatic and covalent contributions [91]. This vision has been confirmed by posterior studies. For instance, Rocha-Rinza et al. used IQA to investigate cooperative effects of hydrogen bonding in small water clusters and also found that the exchange contribution to the interaction energy is particularly strengthened by adjacent hydrogen bond contacts [34]. Additionally, recent work by Rocha-Rinza and coworkers on larger water clusters utilised the ability of IQA to compute the interaction energy of individual contacts to demonstrate that double proton donors and acceptors present cooperativity in addition to anticooperativity and that both phenomena have electrostatic and covalent components [92].

Alkorta et al. compared the hydrogen bonding occurring between charged and neutral carboxylic acid dimers. They found that once the repulsion between the charged groups is discarded, the interactions between the charged molecules resemble those of the neutral ones, thus showing the enduring nature of hydrogen bonds [93].

**Figure 3:**
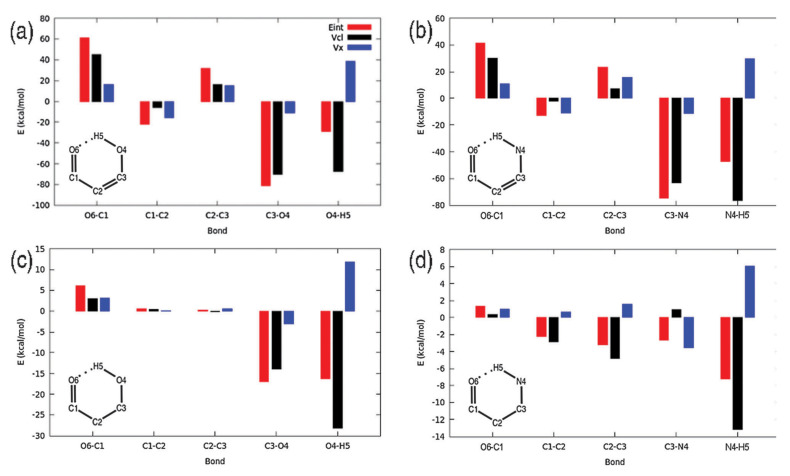
Interacting quantum atoms (IQA) allows for the individual changes in IQA interaction components accompanying the formation of the different hydrogen bonds. Figure from reference [98] shows systems with insaturations and intramolecular HBs (**a**,**b**), along with their non-conjugated counterparts (**c**,**d**). Figure reproduced by permission of the PCCP Owner Societies.

IQA has proven to be particularly useful in the study of systems where a clear partition between different fragments is difficult, as in the case of intramolecular hydrogen bonds. As shown in Figure 3, IQA allows for the calculation of each interaction in a separate way, allowing us to determine which contact is most affected by the formation of any new contact. On this regard, the IQA methodology has been applied by Rocha-Rinza et al. to investigate the origin of the relationship between insaturations and hydrogen bonds [94,95], and the cooperativity and anticooperativity between them [96]. For its part, Eskandari and coworkers have studied intramolecular interaction within vitamin C finding that most conformers are stabilised by cooperative networks of hydrogen bonds [97].

#### 4.4.2. Halogen Bonding

**Figure 4:**
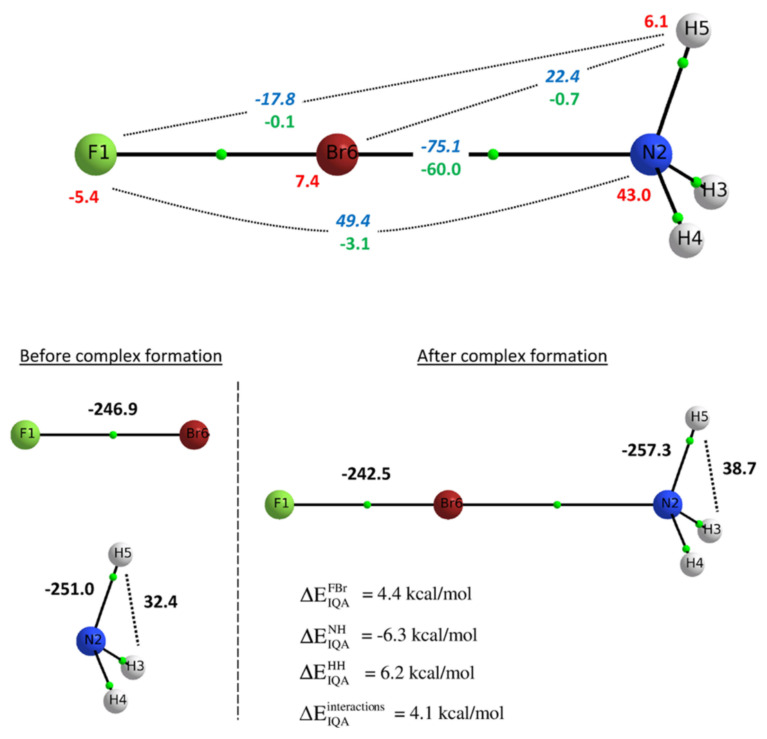
IQA energy partition of the FB_r_ ⋯ NH${}_{3}$ complex. (**Top**) changes intra-atomic energies (red) and intermolecular interactions (classical and exchange terms in blue and green, respectively). (**Bottom**) Change in interatomic energies within each fragment upon complex formation. Figure reprinted (adapted) with permission from reference [98]. Copyright 2020 American Chemical Society.

Peculiar bonding situations have been extensively studied with the help of the IQA methodology. Tognetti and coworkers investigated the nature of halogen bonding in diverse situations such as small complexes [98], intramolecular interactions [99] and halogen-halogen contacts in perhalogenated ethanes [100] Their investigations using DFT densities revealed that for the studied systems the Lewis basis interaction with the halogen atom is always stabilising, being the exchange-correlation the dominant component, with additional attractive electrostatic contributions in most cases. An scheme of this situation is shown in Figure 4. This observation has been confirmed by Popelier et al. in a further study including electron correlation [101].

IQA has been also particularly useful in the characterisation of systems where the categorisation of the interaction as halogen bond is not as straightforward. Eskandari et al., for example, discarded the formation of halogen bonding by fluorine [102]. In contrast, Madzhidov and coworkers found [103] that the interaction between different organoselenium molecules and diiodine indeed complies with the definition of a halogen bond, and Guevara-Vela et al. shown that the dihalogen-water cage interactions in the 5${}^{12}$ and 5${}^{12}$6${}^{2}$ clathrate cages can be considered as halogen bonding [104].

IQA has played an important role in visualising the role of covalency in the halogen-halogen contacts. In the study of bifurcated halogen bonding Bartashevich et al. and Marek et al. found that these types of interaction presents a strong covalent component [105,106]. In an investigation about trihalogen interacting synthons Hariharan and coworkers also highlighted the importance of covalence for these systems [107]. Finally, Duarte et al. studying multicentric halogen bonds shown that strong ionic and covalent contributions are not mutually exclusive [108].

#### 4.4.3. Other Bonding Situations

IQA has also been used to study other chemically interesting bonds. Martín Pendás et al. investigated the chemical bonding in a series of systems that contain beryllium bonds [109], and Casals-Sainz and coworkers applied the IQA methodology to investigate tetrel interactions [110]. In both cases it was shown that the interaction contain important contributions from both its electrostatic and covalent parts.

Madzhidov and Chmutova have employed IQA to study the interaction between dimethylselenide and IIIA group element halides, where the former is an electron pair donor and the later IIIA group atoms act as acceptors. In all the studied complexes the covalence of the Se⋯ A plays a very important role [111].

### 4.5. Bonding of Metallic Elements

The chemical bonding situation in simple transition metal complexes has been also studied using IQA. The first examples of such investigation are studies of metal carbonyls by Martín Pendás et al. which conclude that the metal-ligand interaction in these systems is strongly dominated by covalent effects and that the electrostatic energy associated is comparatively small [112,113]. Other examples of investigations of metal-ligand interactions are the work of Cukrowski and coworkers on the stability of zinc-bipyridil complexes [114] or the study of Albrecht-Schmitt et al. regarding the nature of the fluoride-uranium contact in the (NH${}_{4}$)UF${}_{8}$ crystal [115].

IQA has also been applied to the study of the controversial metal-metal contact. For instance, Tiana et al. demonstrated that in policarbonyl metal clusters the intermetallic interaction is consistent with the description of a delocalised covalent bond that includes both the metals and the carbonyls. However, the global stability of the dimers should be attributed to the electrostatic attraction between the metals and the oxygens and not to this bond [116]. In the intermetallic phase FeGa${}_{3}$ Wagner et al. found that the Fe–Fe interaction energy is negative and therefore stabilising [117].

### 4.6. Organic Chemistry and Reactivity

Taking advantage of its ability to describe systems outside the equilibrium, IQA has been used extensively to study chemical reactions. Jouanno et al. utilised the IQA methodology to quantify the influence of the substituents (through its interatomic interactions) along the reaction path of a thermally controlled metal-free decarboxylative hetero-Diels–Alder (HDA) reaction [118]. Alkorta et al. used IQA to study the mechanism of the adduct formation and capture of CO${}_{2}$ by nitrogen heterocyclic carbenes [119]. In particular IQA allowed them to identify the role played by electrostatic and exchange-correlation factors for different carbenes. Tognetti and coworkers studied the reaction mechanism of a diastereoselective allylation of aldehydes with chiral allylsilanes [120]. Barquera-Lozada investigated the role of different interactions in the torquoselectivity of a series of 3-substituted cyclobutenes [121] and Polo and coworkers studied the reaction mechanism of the oxidative addition of ammonia by Iridium complexes [122]. Alkorta et al. carried out a systematic IQA study of S${}_{N}$2 reactions of the type X${}^{-}$ + CH${}_{3}$X → XCH${}_{3}$ + X${}^{-}$ where X = F, Cl, Br, and I, with particular attention to the the intra-atomic and interatomic energy changes along the reaction path [123].

**Figure 5:**
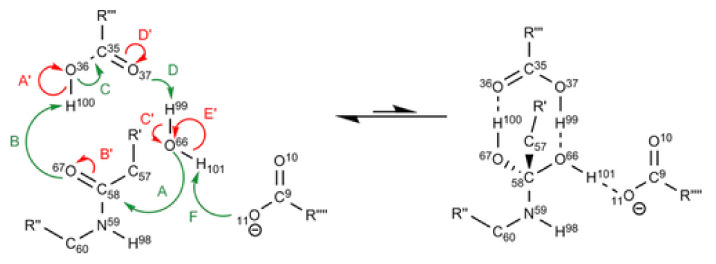
Diagram representing the changes in the strength of the different bonds. The greens arrows represent bond strengthening/forming and while the red ones bond weakening/breaking. Figure taken from Reference [125].

One field where the IQA methodology can be particularly useful is in the examination of catalytic processes, given its ability to account for each individual interaction separately. For instance, Rocha-Rinza et al. have used IQA to investigate the catalytic effect of additional water molecules in the formation of acid rain [124]. Another example is the work of Popelier et al. who studied the mechanism behind peptide hydrolysis in HIV-1 protease [125]. Figure 5 shows parts of this process signalling with different colours bonds that become stronger and weaker.. Through IQA we are able to determine exactly how a specific interaction is affected during a chemical reaction. We believe that as time goes on and IQA gains notoriety, more groups will apply this methodology to catalytic processes.

IQA has also been employed in the study of other phenomena in organic chemistry beyond reactivity and catalysis. For example, Popelier and coworkers studied the *gauche effect*, that is, the atypical situation where a gauche conformation is found to be more stable than the corresponding anti conformation using the IQA methodology. In this investigation it was proposed that the origin of gauche stability is electrostatic polarisation interactions occurring between fluorine atoms [126]. Indeed, IQA has been applied to diverse conformational puzzles—Cukrowski et al. studied the stability of the 2-butene conformers and determined that the origin of the relative energies lies in the interactions between various fragments and cannot be attributed to any specific contact [127], Matczak and coworkers investigated the energetic components governing the conformational behaviour of diheteroaryl ketones and thioketones [128], Vishnevskiy et al. used IQA to explain the relative stability dimethylsubstituted 1,5-diazabicyclo[3.1.0]hexanes [129], Uhlemann and coworkers the explored the interaction in the potential energy surface of the sulfanilamide-water complex, and the sulfanilamide dimer [130] and Maxwell and Popelier determined the cause of the torsional preferences of a series of dipeptides [131].

Passmore and Rautiainen applied the IQA energy partition to compare the Lewis basicity of siloxanes and its analogous ethers. Their analysis revealed that the differences in basicity are related to changes in bonding and polarisation siloxane and diethyl ether in the presence of metal cations. For the former, already polar bonds are further polarised by the metal which generates strong destabilisation which impedes its basicity [132]. Mitzel et al. also used IQA to study pentafluoroethyl-substituted α-silanes concluding that the interactions between the silicon and the donor atoms is mostly an stabilisation effect generated by electrostatics and should not be considered as bonded [133].

Vallejo Narváez et al. used IQA to rationalise that amides dimerise more strongly than imides in spite of their lower acidity [134]. They found that the traditional Jorgensen Secondary Interactions Hypothesis (of repulsion between carbonyl oxygens) fails once the carbon atoms are included in the calculations. Instead, they propose a model based in N-H acidities and C=O basicities, which was later by the same authors for doubly and triply H-bonded complexes consisting in amide/imide homo- and heterodimers and ADA–DAD clusters [135].

### 4.7. Excited State

The IQA methodology is also an useful tool to study systems in the excited states. The first article regarding the use of IQA in the excited state is an investigation of the energetic features of the lowest singlet and triplet states of the H${}_{2}$ molecule carried out by Martín Pendás and coworkers [136]. In spite of this, the application of IQA in the excited state has been limited.

Mosquera an coworkers used this methodology to analyse the $n\to \pi^{*}$ transition in formaldehyde, a paradigmatic example in the discipline. They were able to shown how upon excitation each atom in the CO moiety increases its net energy in detriment of the interaction energy between them [137]. This is in agreement with the common assumption that this the $n\to \pi^{*}$ excitation conducts to the population of an antibonding molecular orbital. Ferro-Costas and coworkers also applied the IQA method to validate the concept of the transferability of functional groups in the excited state [138]. More recently, Hernández-Trujillo et al. completed an investigation of archetypal processes in photophysics from the perspective of the IQA methodology using as examples model reactions. They showed how by using this methodology it is possible to carry out an unified description of these these processes [139].

### 4.8. Steric Repulsion

**Figure 6:**
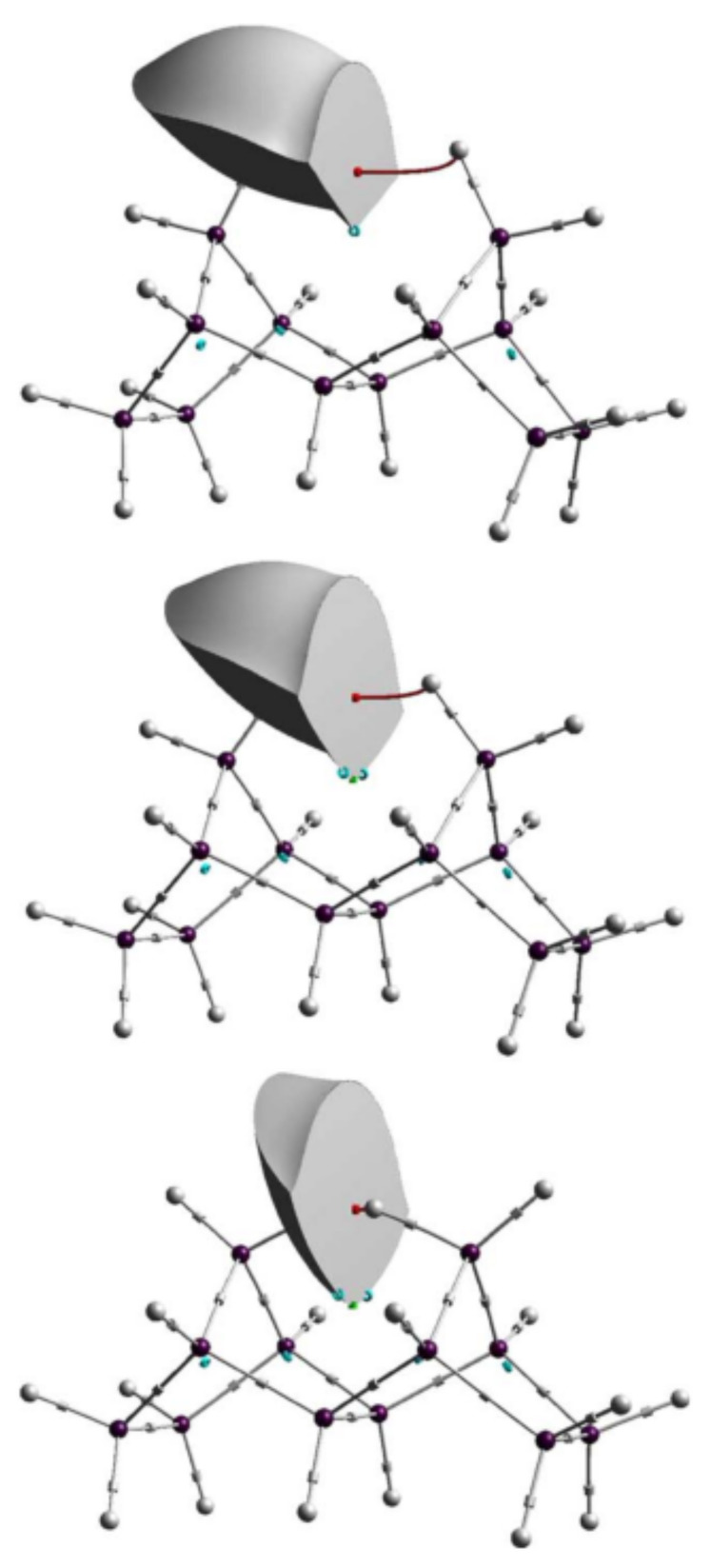
Atomic volume of one of the congested hydrogen atoms in tetra- cyclododecane at a H...H distance of 2.4 Å (**top**), the equilibrium distance of 1.831 Å (**middle**), and 0.5 Å (**bottom**). Figure taken from reference [141].

In a seminal work Pendás et al. explained a series of phenomena, namely steric repulsions, hyperconjugation and stereoelectronic effects, from the perspective of IQA methodology although the main objective of the article was to link IQA concepts with those of EDA [140]. Later on, Dillen delved into the subject, showing how steric hindrance is the result of an increase in the intra-atomic or self-energy of congested atoms [141]. Figure 6 shows how the decrease in the distances between hydrogen atoms modify the volume of the corresponding atoms. Later on, the relationship between congestion and the increase of intra-atomic energy was confirmed by Popelier and coworkers. Moreover they provided evidence of how intraatomic energy can serve in the quantitative description of steric energy [142].

### 4.9. Machine Learning

**Figure 7:**
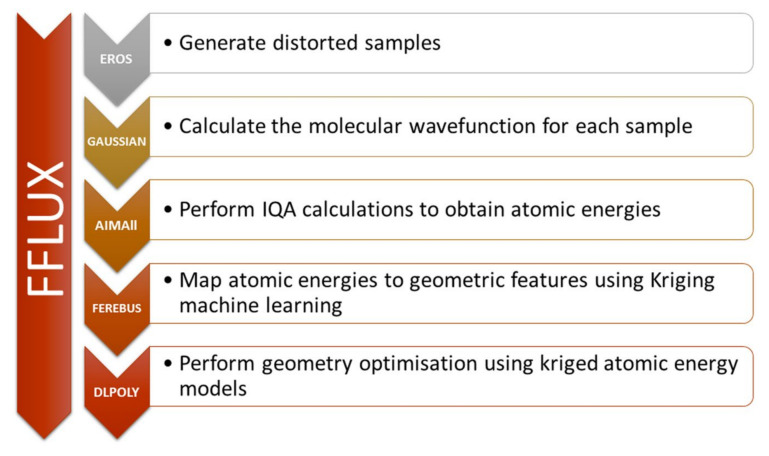
Flowchart representing the different stages in the training (first four steps) and execution (DL_POLY) of the, detailing the programs involved and summaries of their tasks. Figure taken from reference [147].

The research group of professor Popelier have cleverly combined machine learning techniques and the IQA energy partition for a number of applications. Figure 7 shows an scheme of the different steps and programs involved in the training and execution of their models. Using this methodology, they have been able to predict intra- [143] and interatomic energies [144,145], as well as correlation energy at the MP2 approximation for a previously unknown molecular geometries [146] Using. the models produced in this manner, they were able to accurately carry out geometry optimisations with chemical accuracy [147] for biomolecules [148], charged systems [149] and non-covalent interactions [150].

## Figures and Tables

**Table 1:** Structure of the η matrix in a IQA/MP2 calculation. Empty (○) and filled (●) circles represent zero and non-zero values, respectively.

η	*oo* Pairs				*ov* Pairs				*vv* Pairs			
	●	○	⋯	○	○	○	⋯	○	○	○	⋯	○
	○	●	⋯	○	○	○	⋯	○	○	○	⋯	○
	⋮	⋮	⋱	⋮	⋮	⋮	⋱	⋮	⋮	⋮	⋱	⋮
	○	○	⋯	●	○	○	○	○	○	○	○	○
	○	○	⋯	○	●	●	⋯	●	○	○	⋯	○
	○	○	⋯	○	●	●	⋯	●	○	○	⋯	○
	⋮	⋮	⋱	⋮	⋮	⋮	⋱	⋮	⋮	⋮	⋱	⋮
	○	○	⋯	○	●	●	●	●	○	○	○	○
	○	○	⋯	○	○	○	⋯	○	○	○	⋯	○
	○	○	⋯	○	○	○	⋯	○	○	○	⋯	○
	⋮	⋮	⋱	⋮	⋮	⋮	⋱	⋮	⋮	⋮	⋱	⋮
	○	○	⋯	○	○	○	○	○	○	○	○	○
